# Active Packaging Based on Chitosan, Fish Gelatin, Zein, and Kafirin Biopolymers: A Promising Strategy for Innovation in the Cosmetic Sector

**DOI:** 10.3390/polym17243329

**Published:** 2025-12-17

**Authors:** Andres C. Arana-Linares, Alvaro Barrera-Ocampo, Arley Camilo Patiño, Yhors Ciro, Constain H. Salamanca

**Affiliations:** 1 Grupo QBAB, Instituto de Ciencias Aplicadas, Facultad de Ingeniería, Universidad Autónoma de Chile, Av. del Valle Sur 534, Santiago 8580640, Chile; andres.arana@cloud.uautonoma.cl; 2 Grupo Natura, Departamento de Ciencias Farmacéuticas, Biomédicas y Veterinarias, Facultad Barberi de Ingeniería, Diseño y Ciencias Aplicadas, Universidad Icesi, Calle 18 No. 122-135, Cali 760031, Colombia; aabarrera@icesi.edu.co; 3 Departamento de Farmacia, Facultad de Ciencias Farmacéuticas y Alimentarias, Universidad de Antioquia, Calle 70 No. 52-21, Medellín 050010, Colombia; arley.patino@udea.edu.co; 4 Grupo de Investigación en Química y Biotecnología (QUIBIO), Facultad de Ciencias Básicas, Universidad Santiago de Cali, Cali 760035, Colombia; 5 Grupo de Investigación Cecoltec, Cecoltec Services SAS, Cra. 43A #18Sur-135, Medellín 050034, Colombia; 6 Grupo de Investigación Ciencia de Materiales Avanzados, Departamento de Química, Facultad de Ciencias, Universidad Nacional de Colombia Sede Medellín, Cra. 65 #59a-110, Medellín 050034, Colombia

**Keywords:** active packaging, biopolymers, chitosan, cosmetic sector, fish gelatin, kafirin, zein

## Abstract

Background: Biopolymer-based active packaging has experienced significant growth in the food industry due to its capacity to enhance product stability and reduce reliance on synthetic preservatives. However, its application in cosmetics remains limited, despite increasing consumer demand for sustainable and preservative-free solutions. Objective: This review evaluates the feasibility of transferring biopolymer-based active packaging technologies from the food sector to cosmetic applications, identifying relevant materials, processing methods, and implementation challenges. Methodology: A bibliographic search was conducted across nine databases (2000–2025) using the keywords “active packaging,” “antioxidant,” “antimicrobial,” and “biopolymers.” Results: The most recurrent biopolymers identified were chitosan, fish gelatin, zein, and kafirin, all of which exhibit biodegradability, film-forming capacity, and compatibility with natural additives. Although their intrinsic antioxidant and antimicrobial properties are limited, these can be enhanced through the incorporation of bioactive compounds. Processing techniques such as casting, coating, dry forming, and electrospinning were found to be the most effective, enabling customized packaging designs. Key challenges include cost, sensory attributes, mechanical limitations, and regulatory compliance. Conclusion: Active packaging systems based on biopolymers—either alone or combined with natural bioactive ingredients—offer a viable innovation pathway for the cosmetics industry. These systems support clean-label claims and ecological positioning, representing a strategic opportunity to adapt validated technologies from the food sector to meet emerging cosmetic market demands.

## 1. Introduction

Between 2019 and 2024, the global cosmetic and personal care industry experienced a 20% increase in sales, reaching a value of USD 593 billion in 2024 [1]. This growth has underscored its consolidation and relevance in international markets. These products are primarily formulated to enhance physical appearance and promote aesthetic appeal [2], encompassing a wide range of items such as lipsticks, foundations, mascaras, eyeshadows, nail polishes, facial and hand creams, micellar water, sunscreens, serums, and shaving creams. In parallel, personal hygiene has remained a cornerstone of public health, contributing to the prevention of infectious diseases [3]. Daily routines—including bathing, facial cleansing, hair washing, and oral care—have relied on specialized products such as shampoo, conditioner, toothpaste, soap, and mouthwash.

Within the research, development, and innovation (RDI) landscape, cosmetic formulations are required to meet stringent quality and safety standards to ensure their commercial viability. Among these, product stability has been recognized as a critical criterion, as it guarantees the preservation of active ingredients throughout the intended shelf life [4]. To achieve this, preservatives are commonly incorporated—compounds capable of inhibiting microbial growth and mitigating oxidative degradation and the formation of reactive oxygen species (ROS) [5,6]. Widely used preservatives include parabens [7,8], aldehydes and formaldehyde donors [9,10,11,12], organic acids [13,14], isothiazolinone [15,16], alcohols, and phenols [17,18]. In addition to preservatives, packaging materials play a pivotal role in maintaining product integrity. These are typically manufactured from synthetic polymers such as polyethylene, polypropylene, polymethylmethacrylate, nylon, silicon, terephthalate, and high- or low-density polyethylene [2,19,20,21].

However, both preservatives and conventional packaging materials have raised concerns regarding human health and environmental safety. Formaldehyde has been classified as carcinogenic by the International Agency for Research on Cancer (IARC) [22], while parabens are suspected endocrine disruptors [23,24]. Moreover, the environmental burden associated with non-biodegradable synthetic polymers has intensified the search for safer and more sustainable alternatives.

In response to growing consumer awareness of ingredient safety and environmental sustainability, the cosmetics industry has begun to explore innovative strategies for formulation preservation and packaging. Among these, active packaging has emerged as a promising alternative. Defined as systems that not only contain and protect a product but also interact with it or its environment in a controlled manner, active packaging aims to enhance product quality, stability, and shelf life [25,26,27]. Unlike conventional packaging, which serves a passive role, active packaging can release beneficial agents such as antioxidants or antimicrobials, or absorb undesirable compounds like oxygen, moisture, or free radicals [28,29,30].

Over the past decade, significant advances have been reported in the development of active packaging, particularly within the food sector. For instance, chitosan films incorporating calcium oxide have demonstrated antimicrobial activity against *E. coli* and *S. aureus* [31]; nanocellulose-based coatings with selenium nanoparticles have exhibited high antioxidant capacity [32]; and polyvinyl alcohol films embedded with carbon quantum dots from green tea extracts have provided UV protection and enhanced oxygen barrier properties [33]. These systems have proven effective in extending shelf life and preserving the quality of perishable foods, thereby consolidating the technological potential of active packaging.

Although its application in cosmetics remains limited, the transfer of active packaging technologies from food to cosmetic sectors has gained relevance. Many cosmetic formulations contain oxidation-sensitive compounds—such as oils, fats, and antioxidants—and are susceptible to microbial contamination [34,35,36]. Under these conditions, active packaging offers a viable strategy to reduce reliance on synthetic preservatives, improve physicochemical and microbiological stability, and meet consumer demand for safer, more sustainable, and skin-compatible products.

To support this perspective, a broad literature review was conducted across multiple databases (2000–2025), focusing on keywords such as “active packaging,” “antioxidant,” “antimicrobial,” and “biopolymers.” This exploratory search aimed to identify the most recurrent materials and processing techniques relevant to active packaging, without following a formal systematic review protocol. Chitosan, fish gelatin, zein, and kafirin emerged as key biopolymers with transferable potential from food to cosmetic applications. Their functional versatility, biodegradability, and compatibility with natural additives position them as strategic candidates for innovation in cosmetic packaging—a domain still underexplored in the current literature.

## 2. Biopolymers Frequently Used in the Development of Active Packaging

Active packaging materials often incorporate bioactive agents such as antimicrobials, antioxidants, and moisture absorbers, which may be embedded within the packaging matrix or contained in separate sachets, working synergistically to control microbial growth, oxidative processes, and environmental conditions [28]. Within this framework, several natural polymers have garnered particular attention due to their biocompatibility, biodegradability, and functional versatility. Notably, chitosan, fish gelatin, and vegetal proteins such as zein and kafirin have been extensively studied as matrix materials for active packaging applications. These biopolymers not only offer film-forming abilities and barrier properties but also serve as carriers for incorporating active compounds [37], thus contributing to the extended shelf life and improved safety of cosmetic products.

### 2.1. Chitosan

**Figure 1:**
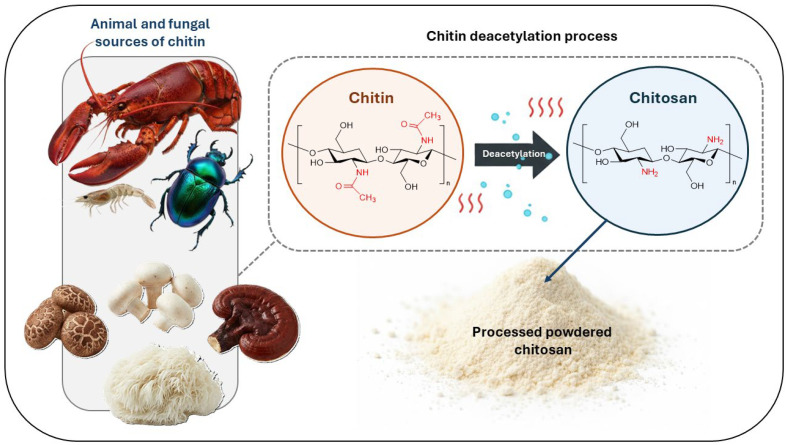
Schematic representation of the chemical conversion of chitin to chitosan by deacetylation. The acetyl moieties (–COCH_3_) highlight-ed in red on the chitin repeating units are cleaved during processing, resulting in the exposure and formation of free amino groups (–NH_2_), highlighted in red on the chitosan backbone. Chitin is sourced from animal exoskeletons and fungal cell walls, while chitosan is commonly processed into powders and films for diverse industrial applications.

Chitin is recognized as one of the most abundant biopolymers in nature, second only to cellulose. It is present in the exoskeletons of crustaceans and insects, as well as in the cell walls of several fungi, where it fulfills a fundamental structural role [38,39,40,41]. Its wide availability and renewable nature have established chitin as a strategic resource for obtaining derivatives of interest in industrial fields. Among these derivatives, chitosan is the most notable, as it is produced through the deacetylation of chitin (Figure 1) and combines unique properties that position it as a highly versatile material [42,43,44,45,46,47,48].

The key step in the production of chitosan from chitin is the removal of acetyl groups from the polymeric chain, a process that significantly increases the number of free amino groups (-NH_2_) (Figure 1). This structural modification not only enhances the solubility of the polymer in acidic aqueous environments but also confers a polycationic nature, which is responsible for many of its biological and technological interactions [49]. The degree of deacetylation (DD) is considered a critical parameter, as it determines important physicochemical properties such as charge density [50], solubility [51], antimicrobial activity [52], and the ability to form interactions with metal ions [53]. However, achieving a high DD remains a major challenge in industrial-scale production, since it requires precise control of pH, temperature, and processing time. Strict conditions can lead to polymer degradation and the generation of undesirable byproducts, which reduce the overall yield and compromise product quality [54].

To overcome these limitations, several technological alternatives have been explored with the aim of achieving a balance between efficiency, sustainability, and product quality. Autoclave-assisted deacetylation has been reported to shorten reaction times and achieve DD values close to 83%, with high yields and reduced polymer degradation [54]. Microwave-assisted synthesis can increase DD values to more than 90%, producing chitosan with improved thermal stability, higher crystallinity, and reduced molecular weight [55], which are desirable characteristics for specific applications.

On the other hand, chitosan has been classified according to its molecular weight into low (below 150 kDa), medium (150–750 kDa), and high (above 750 kDa) ranges [56]. In particular, lower molecular weight confers markedly enhanced solubility and reduced solution viscosity, facilitating the fabrication of thin, uniform, and mechanically stable films [57,58]. Additionally, shorter chitosan chains diffuse more efficiently and interact more effectively with target surfaces, thereby strengthening antimicrobial performance and enabling the development of high-functionality coatings with superior protective properties [59,60]. Enzymatic methods are also emerging as promising eco-efficient alternatives, as they allow the production of chitosan of comparable quality to that obtained by conventional processes, while simultaneously optimizing upstream steps such as deproteinization and demineralization of crustacean shells [61]. These advances highlight the transition toward more sustainable and efficient production processes, in accordance with the current demands of biotechnology and material sciences.

The structural characteristics of chitosan give rise to a particular molecular behavior that supports its technological applications. The rigidity of its polymeric chains, which results from equatorial glycosidic linkages, is combined with the ability to extend under protonated conditions, driven by the repulsion between positively charged amino groups [62]. This structural dynamism allows chitosan to adapt to different environments and provides considerable versatility in its applications.

From a functional perspective, three key properties are especially relevant: the ability to form complexes with transition metals, pH-dependent solubility, and charge density. First, chitosan can interact with cations such as Cu^2+^, Zn^2+^, and Ni^2+^ [63,64,65], with copper binding being particularly significant in the context of active cosmetic packaging, where the controlled release of ions contributes to prolonged antimicrobial activity [66]. Second, its solubility manifests primarily in acidic environments with pH values below 6.0, where the protonation of amino groups facilitates polymer dispersion in solution [67]. Finally, the charge density, which is determined by the DD and influenced by factors such as pH and ionic strength [68], is directly associated with the antimicrobial potential of the material, since it regulates the interactions of chitosan with cell membranes and biomolecules [69].

Beyond its intrinsic properties, chitosan has emerged as a versatile matrix for the incorporation of natural bioactive compounds. This is particularly relevant for the development of active packaging in the cosmetic industry, where functional ingredients such as oils, lipids, and functional ingredients are highly susceptible to degradation processes induced by ROS, light, temperature fluctuations, and microbiological contamination [70]. The incorporation of flavonoids, phenolic acids, tannins, and terpenoid-rich plant extracts into chitosan-based matrices not only enhances the antioxidant and antimicrobial potential of the material but also contributes to preserving the physicochemical and microbiological stability of cosmetic formulations. Owing to these distinctive features, chitosan has attracted significant attention as a polymeric matrix for the development of functional films in cosmetic packaging. Its biodegradability, biocompatibility, and versatility make it a renewable alternative to conventional synthetic materials, while its intrinsic bioactivity provides added value for the preservation of functional cosmetic ingredients. Rather than serving solely as a passive barrier, chitosan-based packaging materials actively extend product shelf life and reduce the need for synthetic additives, positioning them as a highly promising strategy for next-generation active packaging systems [71,72].

### 2.2. Fish Gelatin

Gelatin is a natural biopolymer derived from collagen, a protein consisting of three chains with different molecular weights after hydrolysis, α-chain (80–125 kDa), β-chain (160–250 kDa), and γ-chain (240–375 kDa), along with low-molecular-weight substances such as mineral salts and water [73]. In relation to fish gelatin, it is worth highlighting that its use has increased significantly as an alternative to conventional gelatin of bovine or porcine origin, mainly due to the large quantities of skins and scales generated as by-products of the fishing and aquaculture industries, which makes this material more readily available and sustainable. In addition, the extraction and purification processes of fish gelatin are generally simpler and less costly than those required for traditional sources, favoring its application in the development of bioactive biopolymer-based packaging systems and contributing to the valorization of marine by-products, as widely documented in the literature [74]. Beyond its availability, fish gelatin exhibits distinctive physicochemical characteristics—including lower gelation and melting temperatures [75,76], higher solubility [77], and a unique amino acid profile [78,79]—that directly influence its rheological and mechanical behavior, making it particularly suitable for use in heat-sensitive pharmaceutical and cosmetic formulations.

Gelatin is commonly classified into two main types according to the pretreatment applied to collagen: type A (acid-processed) and type B (alkaline-processed). In addition, enzymatic technologies have been developed to hydrolyze collagen fibers with greater selectivity and control [80].

**Figure 2:**
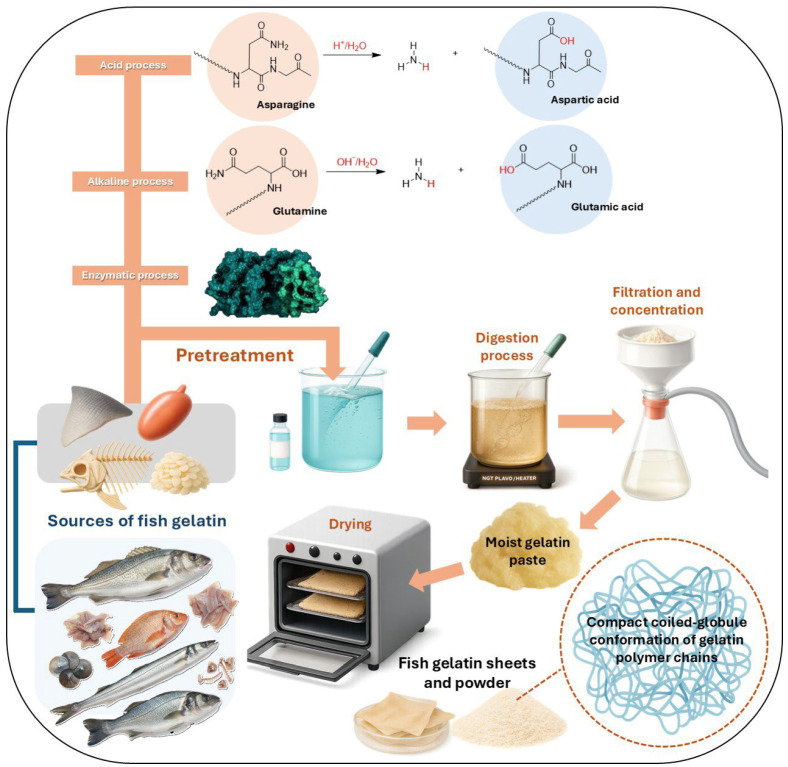
Fish gelatin extraction: pretreatment technologies and collagen network structure. Gelatin is extracted from fish by-products such as bones, skin, and scales through three main pretreatment strategies: acidic, alkaline, and enzymatic processes. As highlighted in red, the acidic and alkaline treatments promote the deamidation of asparagine and glutamine residues into aspartic acid and glutamic acid, respectively, through hydrolytic reactions (H^+^/H_2_O and OH^−^/H_2_O). The molecular network structure obtained after extraction is highlighted, depicting the interconnected collagen-derived chains characteristic of gelatin.

Fish gelatin is obtained by processing collagen extracted from fish by-products such as skin, scales, and bones through controlled hydrolysis (Figure 2). During this conversion, significant structural modifications occur, including the deamination of asparagine to aspartic acid in acid treatments and the deamination of glutamine to glutamic acid in alkaline treatments, resulting in a partially degraded structure compared to native collagen [81,82]. Among these materials, type A fish gelatin exhibits particularly advantageous structural and functional characteristics for the development of active bio-based packaging, especially for solid cosmetic products. The acid-induced conversion of asparagine to aspartic acid increases the density of carboxyl groups, enhancing hydrophilicity, solubility, and film-forming capacity [83,84]. These attributes enable the fabrication of homogeneous, flexible, and functional films capable of incorporating antioxidants, antimicrobials, or other active agents, making type A gelatin highly suitable for active packaging systems [85,86,87]. Nevertheless, its enhanced solubility and hygroscopicity impose limitations for liquid or high-moisture formulations, where premature dissolution and loss of mechanical integrity may occur. Considering both its functional advantages and application-specific constraints, type A fish gelatin provides a strategically valuable biomaterial for biodegradable, thermo-sensitive, and active biopackaging in solid cosmetic products. For these reasons, and due to its relevance to current technological demands, the present review focuses specifically on type A fish gelatin.

Type A gelatin is characterized by an isoelectric point within the pH range of 6 to 9, a consequence of the applied acid treatment. These gelatins exhibit physicochemical properties advantageous for producing bio-based packaging materials, including gel strength, viscosity, melting temperature, and the α/β chain ratio. Specifically, fish gelatin has reported gel strengths between 70 and 270 bloom, with gelation and melting temperatures ranging from 8–25 °C and 11–28 °C, respectively [88,89,90]. Its notable hydrophilicity and high solubility in cold water facilitate processing in aqueous systems, which is favorable for developing environmentally friendly and sustainable polymeric materials.

Moreover, fish gelatin stands out due to its biodegradability and biocompatibility, positioning it as a promising alternative to synthetic polymers and traditional mammalian gelatins, particularly in the sector of biodegradable active packaging [76]. However, it displays lower mechanical properties and structural stability under usage conditions compared to mammalian gelatins, which poses challenges for commercial applications [91]. To overcome these limitations, composite films combining fish gelatin with other polymers, such as chitosan, have been explored to enhance mechanical strength and water vapor barrier properties [92,93], thereby improving their potential functionality in cosmetic packaging applications.

Gelation temperature, melting temperature, and gel strength are the most critical functional and technical properties of gelatin that determine its commercial utility. These properties are primarily influenced by gelatin molecular weight and its internal amino acid composition. Fish gelatin, in comparison to mammalian gelatin, contains lower levels of proline and hydroxyproline, key amino acids crucial for forming the collagen-like triple helix structure [93]. This deficiency accounts for the generally lower gel strength and melting temperature observed in fish gelatin. Moreover, gel strength correlates positively with the content of α-chains, while a lower α/β chain ratio negatively impacts its functional properties [94,95]. These inherent characteristics restrict the broad replacement of mammalian gelatin by fish gelatin in packaging technology applications, where mammalian gelatin typically exhibits superior thickening, stabilizing, and gelling capabilities [91]. Nevertheless, fish gelatin remains a promising alternative, provided that its structural and thermal particularities are addressed through tailored technological approaches to achieve functional equivalence—an effort that is further supported by its inherently favorable film-forming properties and high compatibility with natural bioactive compounds [96]. Recent studies have focused on the incorporation of plant- and biomass-derived additives into fish gelatin matrices to enhance their mechanical, barrier, and functional properties. The combination of gelatin with polyphenols, essential oils, polysaccharides, and nanostructured materials has demonstrated significant improvements in stability, antioxidant activity, and antimicrobial efficacy. These composite films have been successfully applied in food preservation, showing extended shelf life and protection against microbial contamination and lipid oxidation [97,98,99]. Such findings highlight the potential translation of fish gelatin-based films into the cosmetic sector, where they could serve as active or intelligent packaging materials to protect sensitive formulations, such as emulsions, creams, oils, and gels, from degradation and microbiological contamination.

### 2.3. Plant-Based Proteins (Zein and Kafirin)

**Figure 3:**
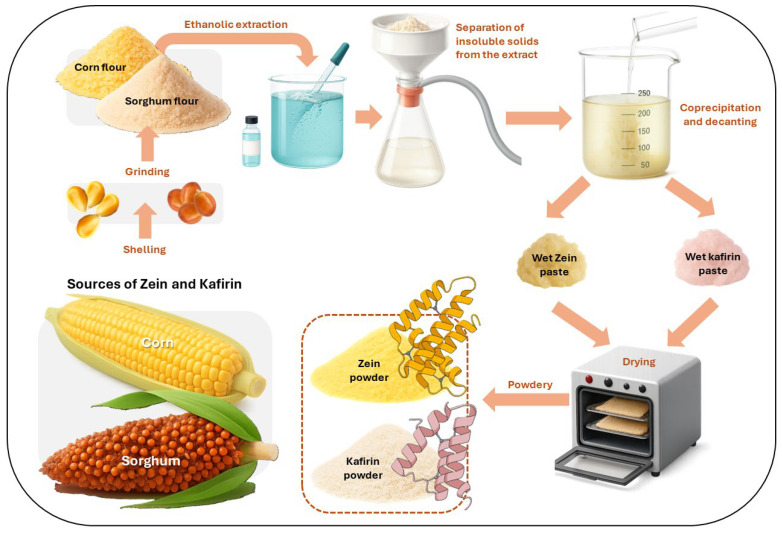
Zein and Kafirin Extraction. Corn and sorghum grains are processed and extracted with ethanol. Filtration and drying yield protein powders. The α-helix-rich models of zein and kafirin were generated using Copilot for illustrative purposes.

Among the plant-based proteins with growing interest for active packaging in cosmetics, zein and kafirin stand out as emerging biopolymers with promising functional potential. Zein is the main storage protein of maize endosperm and a by-product of the bioethanol industry, which makes it an attractive, sustainable raw material [100]. Its extraction process from corn and the subsequent recovery of zein powder through ethanolic extraction, filtration, and drying are schematically illustrated in Figure 3. It belongs to the prolamin family and is characterized by its hydrophobic nature, being insoluble in water at pH < 12 due to its high content of non-polar amino acid residues. This protein is considered non-toxic and exhibits favorable properties such as low permeability, thermal resistance, and intrinsic antimicrobial activity, which have supported its use in edible coatings for food and pharmaceutical products. Laboratory-scale studies have demonstrated successful processing of zein films through casting, extrusion, and co-extrusion techniques [101,102]. From a compositional perspective, zein consists of four fractions (α, β, γ, and δ) with differences in molecular weight and amino acid polarity. The α (19–22 kDa), γ (27 kDa), and δ (10 kDa) fractions are predominantly apolar, while the β fraction contains a more balanced ratio of polar and apolar residues [103]. Glutamic acid is the most abundant amino acid (≈27.5%), followed by proline, alanine, aspartic acid, and phenylalanine [104]. Although largely hydrophobic, zein also contains polar and basic residues such as arginine and histidine, enabling electrostatic interactions with acidic groups and explaining its solubility in mixed solvents rather than in pure water or ethanol [103].

Similarly, kafirin, the major storage protein of sorghum (*Sorghum bicolor*), accounts for 60–80% of the total grain protein and exhibits close structural similarity to zein, though with an even higher degree of hydrophobicity [105]. This property contributes to the formation of more stable films, making kafirin an equally interesting candidate for packaging applications. However, its extraction requires hydroalcoholic processes that yield precipitates containing residual triglycerides (≈8%) and free fatty acids (≈1%), which must be removed to obtain functional biopolymer matrices. The properties of kafirin-based films depend on multiple factors, including protein purity, solvent system, pH, plasticizers or additives, drying temperature, and ambient humidity [106]. Structural organization also plays a critical role: films dominated by α-helical conformations exhibit higher tensile strength, improved rupture resistance, and lower water vapor permeability (Figure 3), whereas those with a predominance of β-sheet structures display weaker mechanical and barrier performance [107,108]. At the molecular level, kafirin is classified into α-, β-, γ-, and δ-fractions according to amino acid composition, solubility, and electrophoretic mobility. These fractions differ in length and residue distribution: α-kafirin (240–250 mainly non-polar residues, including lysine and tryptophan), β-kafirin (172 residues, including Met, Cys, Trp), γ-kafirin (193 residues, including Pro, Cys, His, Lys, Asn, Trp), and δ-kafirin (114 residues, including Met, Lys, Trp) [106,109]. Due to its highly apolar nature, kafirin exhibits limited solubility in conventional solvents; nonetheless, aqueous ethanol and acetone, particularly under elevated temperatures, have proven effective for solubilization and film formation. Additionally, Oguntoyinbo, et. al. demonstrated that food-compatible solvents such as acetic and lactic acids at 70 °C efficiently dissolve kafirin, reflecting its amphoteric character [110]. Zein and kafirin illustrate the versatility of plant-derived prolamins as functional biopolymers for cosmetic packaging. Their intrinsic hydrophobicity, strong film-forming capacity, and adaptability to diverse processing techniques enable the development of biodegradable materials with tailored barriers, mechanical, and bioactive properties.

Taken together, the analysis of chitosan, fish gelatin, and plant-derived prolamins such as zein and kafirin highlights that each biopolymer presents a distinctive combination of functional, structural, and processing-related attributes that directly influence its suitability for cosmetic packaging applications. While some materials excel in film-forming capacity or intrinsic bioactivity, others offer superior hydrophobicity or barrier performance but face challenges related to mechanical stability, solubility, or scalability. Importantly, many of these limitations can be strategically mitigated through blending, plasticization, crosslinking, or incorporation of complementary polymers—such as starch—allowing the design of optimized composite systems tailored to specific cosmetic formulations. In this context, a comparative assessment becomes essential to rationally select materials according to the functional requirements of the final application. The main advantages and limitations of the biopolymers studied in this review—chitosan, fish gelatin, zein, kafirin, and starch—are therefore summarized in Table 1, highlighting their potential and constraints for cosmetic packaging applications.

### 2.4. Moisture Sensitivity and Functional Suitability of Biopolymers for Cosmetic Packaging

Despite their widespread use as matrix materials for active packaging, the intrinsic interaction of biopolymers with water is a critical factor determining their suitability for different cosmetic formulations. Chitosan and fish gelatin, for example, exhibit pronounced water uptake and high moisture permeability due to their hydrophilic functional groups and the capacity of their polymer chains to swell, hydrate, and even undergo partial solubilization in aqueous environments [49,50,51,77]. When these materials encounter liquid formulations, such as emulsions, aqueous gels, tonics, or other high-moisture cosmetic systems, they may lose structural integrity through excessive swelling, hydrogel formation, or dissolution [111,112,113,114]. These limitations restrict their direct use as primary packaging for liquid cosmetics unless specific technological reinforcements—such as chemical or enzymatic crosslinking, hydrophobic polymer blending, lamination with water-resistant layers, or the application of surface coatings—are incorporated to enhance moisture resistance and stabilize the packaging matrix. For example, several studies have shown that blending gelatin with more water-resistant biopolymers can markedly improve its stability: chitosan incorporation strengthens the matrix, reduces solubility, and lowers water-vapor permeability by up to 50% [92], while hydrophobic zein increases crystallinity, raises contact-angle values, and decreases film solubility to about 40%, enhancing moisture tolerance [115]. These results demonstrate how polymer reinforcement strategies can effectively mitigate the inherent water sensitivity of gelatin-based materials.

In contrast, plant-derived prolamins such as kafirin and zein exhibit markedly lower affinity for water due to their hydrophobic and non-polar amino acid composition, making them considerably more stable in the presence of semi-solid or moderately hydrated cosmetic formulations, including sticks, balms, solid conditioners, dry masks, or anhydrous pastes [106,109]. Nonetheless, even these comparatively water-resistant biopolymers may require structural reinforcement, plasticization, or nanocomposite engineering to meet specific mechanical, permeability, and durability requirements. For example, tannin-modified kafirin films exhibit pronounced mechanical reinforcement—showing up to two- to four-fold increases in tensile strength and Young’s modulus, along with reduced water uptake—while LAPONITE^®^-based kafirin nanocomposites displayed enhanced rigidity and structural stability without detrimental changes in water-vapor permeability [116,117]. Likewise, the incorporation of TiO_2_ nanoparticles into zein matrices strengthens tensile performance, improves thermal stability, and decreases both oxygen and water-vapor permeability by reducing porosity and consolidating the internal microstructure [118]. Together, these considerations highlight the need to match the physicochemical profile of each biopolymer with the moisture content, rheological characteristics, and stability requirements of the targeted cosmetic product when designing bio-based packaging systems.

## 3. Methods for the Development of Active Packaging

**Figure 4:**
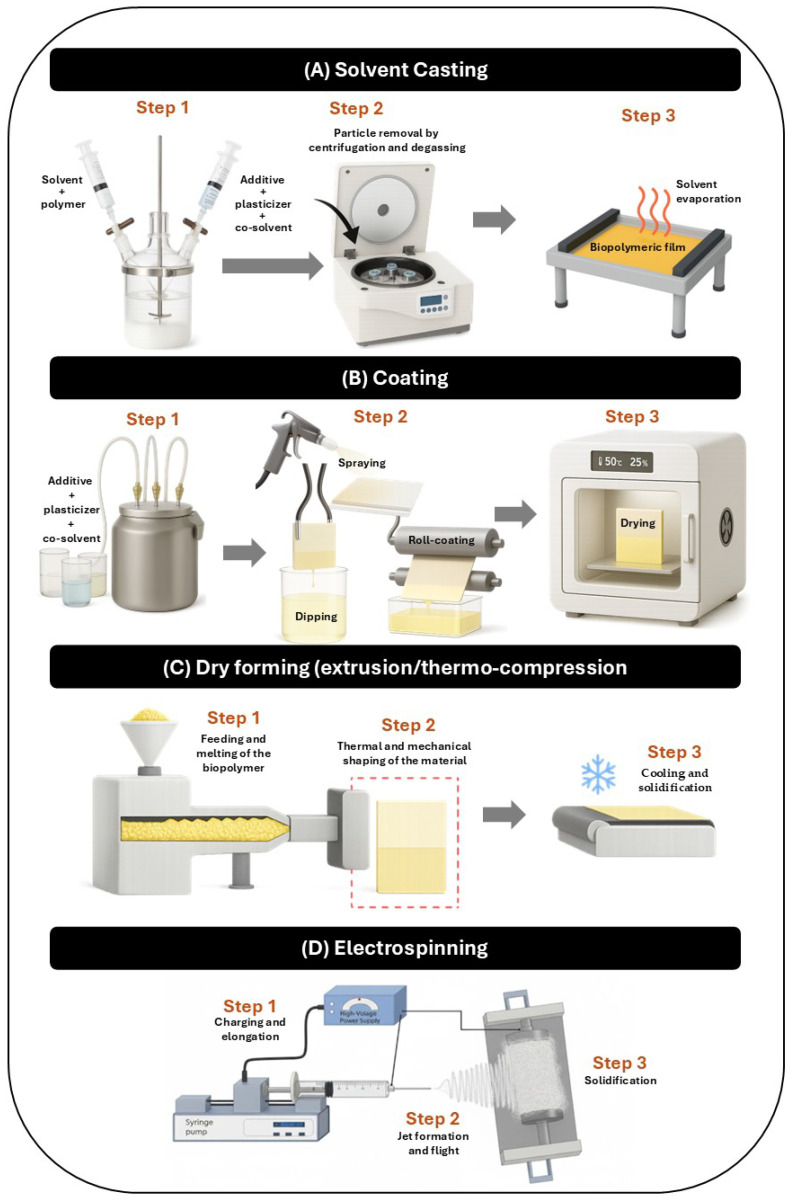
Biopolymer processing methods for active packaging—(**A**) casting, (**B**) coating, (**C**) dry forming, (**D**) electrospinning—each shown in preparation, application, and solidification steps.

Active packaging increasingly uses biopolymers with antioxidant or antimicrobial properties, alone or combined. Techniques like solution casting and surface coating are common for film and coating formation. Emerging methods such as dry forming and electrospinning enable tailored microstructures and enhanced performance. Figure 4 compares four approaches—casting, coating, dry forming, electrospinning—across preparation, application, and solidification.

### 3.1. Solvent Casting

Solvent casting is one of the most widely employed laboratory-scale techniques for the fabrication of biopolymer-based films, particularly for hydrophilic polymers such as gelatin, chitosan, starch and cellulose derivatives (Figure 4A). The process involves dissolving the biopolymer in a suitable solvent—typically water, dilute acetic acid (for chitosan), or hydroalcoholic mixtures—followed by the incorporation of plasticizers (e.g., glycerol, sorbitol) and optional additives or secondary polymers. The solution is then homogenized, degassed and cast onto leveled substrates (glass, Teflon or polyester plates), where solvent evaporation under controlled temperature (20–50 °C) and relative humidity (40–60%) yields a continuous film within 24–48 h [119].

Films produced by solvent casting typically exhibit thicknesses ranging is commonly reported in the range of tens to a few hundred micrometers. For chitosan, Eulalio et al. prepared films of about 100 µm thickness from chitosan solutions in different acids, while more recent work on chitosan packaging films reports thickness values around 0.05 mm (~50 µm) [120,121]. Fish-gelatin films obtained by casting skin-gelatin solutions usually show thicknesses between ~0.05 and 0.15 mm; for example, Avena-Bustillos et al. evaluated water-vapour permeability on 0.05-mm fish-gelatin films, and Pouralkhas et al. reported fish-gelatin/fucoidan films with average thicknesses ranging from 0.12 to 0.147 mm, while other studies describe ranges of 0.051–0.069 mm depending on formulation [122,123,124]. Zein films produced by ethanol-based solvent casting have also been measured in the ~0.06–0.22 mm range, with thin films of ~50 µm used in dynamic mechanical analysis and thicker films (0.14–0.22 mm) reported for plasticized zein formulations [125,126]. Kafirin films obtained by casting in organic acids are described as very thin (<50 µm) or prepared with a typical film thickness of about 100 µm in comparative studies of zein, kafirin and avenin films [127]. Overall, these data indicate that for chitosan, fish gelatin, zein and kafirin, solvent-cast films are commonly engineered with thicknesses in the ~50–150 µm range, which offers a practical compromise between mechanical integrity, flexibility and barrier performance for packaging applications.

Compared with conventional packaging materials, solvent-cast films often exhibit superior antioxidant and antimicrobial performance, chiefly due to their specific formulation (active compounds and biopolymer composition) rather than the casting technique itself, as shown by studies confirming their effectiveness in preserving product quality [128,129,130]. This technique is especially suitable for incorporating thermosensitive and volatile bioactive compounds, as it operates at low temperatures and under mild shear conditions, thereby minimizing chemical and structural degradation of both the polymer matrix and the active ingredients [131,132]. Nevertheless, important limitations such as poor scalability, prolonged drying times, potential solvent residues, and batch-to-batch variability remain critical challenges [133,134,135]. These drawbacks can be partially mitigated through the application of cross-linking strategies (chemical or enzymatic), polymer blending approaches (e.g., gelatin–chitosan or gelatin–zein systems), and the use of surface coatings or lamination with hydrophobic outer layers, which contribute to improved mechanical strength and enhanced moisture barrier properties [136,137,138,139].

### 3.2. Coating

Coating represents a versatile strategy to functionalize the surface of primary packaging materials by depositing a thin biopolymer layer onto a preformed substrate (Figure 4B). Typically, a polymer solution or dispersion containing the biopolymer (e.g., gelatin, chitosan), plasticizers, and functional additives is applied onto the surface by spraying, dipping, roll-to-roll or slot-die coating, followed by controlled drying to form a continuous film [140].

Unlike free-standing films, coated layers based on chitosan, fish gelatin, zein or kafirin are typically much thinner, with representative systems showing ultrathin chitosan layers of ~1–2 µm on eggshells and up to ~10 µm after multiple applications, chitosan– and fish-gelatin–based coatings on fresh-cut apples in the range of ~35–60 µm, and zein coatings on tomatoes with controlled thicknesses of approximately 5, 15 and 66 µm [141,142,143].

However, the same feature that makes these coatings attractive from a material- and functionality-efficiency perspective—their extremely reduced thickness—also becomes their main structural limitation, significantly increasing their susceptibility to cracking, delamination and moisture-induced degradation, as commonly observed in ultra-thin barrier layers and biopolymer coatings under humid conditions [144,145,146]. Key challenges include the low mechanical resistance of ultra-thin layers, the formation of cracks and defects, and the limited durability of hydrophilic matrices at high relative humidity [146,147]. These limitations can be mitigated or overcome by designing multilayer architectures, using compatibilizing blends and cross-linking agents, and incorporating hydrophobic components such as waxes or zein, which improve moisture barrier and structural integrity [148,149,150,151]. In addition, post-treatment strategies based on plasma activation or UV-curing have been shown to enhance coating adhesion, reduce delamination and extend the functional lifetime of thin chitosan- and protein-based layers on polymeric substrates [137,152,153].

### 3.3. Dry Forming (Extrusion and Thermo-Compression)

Dry-forming processes, including extrusion and thermo-compression, are solvent-free techniques that rely on polymer melting or thermal softening followed by shaping under pressure and subsequent cooling to obtain continuous films or sheets (Figure 4C). When applied to biopolymers such as zein and kafirin, which exhibit more hydrophobic and thermoplastic behavior [154,155], these methods can produce films with typical thicknesses ranging from 100 to 500 μm, and up to approximately 1 mm in more rigid structures [156,157]. In contrast, the use of chitosan and fish gelatin in these processes generally requires prior thermoplasticization and the incorporation of plasticizers (e.g., glycerol, sorbitol or polyethylene glycol) to enable adequate flow and prevent excessive brittleness [158,159,160].

Due to the high processing temperatures (approximately 80–200 °C) required in these techniques, their compatibility with volatile and thermosensitive active compounds is limited [159]. Moreover, in protein-based biopolymers such as fish gelatin and kafirin, excessive thermal exposure may lead to denaturation, chain scission, oxidation or loss of functional groups, resulting in reduced film flexibility and altered barrier properties [161,162,163,164]. Consequently, sensitive cosmetic actives (e.g., essential oils, vitamins, phenolic antioxidants) are preferably microencapsulated prior to processing or incorporated post-extrusion via surface coating or lamination [165,166,167,168,169,170].

Despite these limitations, hot-melt extrusion (HME) has gained increasing relevance in the development of bioactive protein-based packaging, particularly in thermoplastic zein systems, as it enables precise control over film thickness, density, mechanical strength and gas/moisture permeability [156,171,172,173,174]. Kafirin-based films similarly exhibit promising oxygen and water vapor barrier properties and can be formulated as bioactive matrices incorporating antimicrobial and antioxidant agents [175,176]. Furthermore, the integration of functional modifiers—such as enzymatic cross-linkers, hydrophobic additives and nanostructured reinforcements including cellulose nanocrystals or layered silicates—has been shown to improve flexibility, enhance antimicrobial performance and optimize the barrier properties of zein- and kafirin-based films [177,178,179,180,181,182].

### 3.4. Electrospinning

Electrospinning is a high-voltage technique that enables the production of ultrafine fibrous films and coatings with tunable morphology and porosity (Figure 4D). It involves the ejection of a charged polymer jet from a Taylor cone, followed by fiber stretching and solvent evaporation before deposition on a grounded collector [183,184,185].

This method typically produces nanofibrous mats with fiber diameters ranging from 50 nm to several micrometers and overall film thicknesses between 10 and 230 μm, depending on deposition time [186,187,188,189,190]. Electrospinning is highly compatible with thermosensitive, volatile and bioactive compounds, since it operates at ambient temperature and allows incorporation via blending, coaxial or emulsion electrospinning [191,192,193].

Biopolymers such as gelatin, chitosan, and zein have been successfully electrospun into highly porous, high-surface-area structures. Although this porosity precludes their use as stand-alone barrier films, it enables their application as functional layers in multilayer systems, where they regulate permeability and serve as efficient carriers for antioxidant and antimicrobial agents [194,195,196,197].

Limitations include low large-scale productivity, dependence on solvent systems and potential structural instability under high humidity [198,199,200,201,202]. These challenges can be addressed by crosslinking, multilayer electrospinning, blending with hydrophobic polymers or post-treatment (e.g., UV, thermal or chemical stabilization) [203,204,205,206].

Electrospun materials have also been explored as preservative-free cosmetic delivery systems, such as antioxidant-enriched dry facial masks activated by moisture, providing multifunctional performance (hydration, antioxidant and antimicrobial effects) [207].

### 3.5. Other Emerging Methodologies

Beyond conventional approaches, several emerging techniques have expanded the design space for functional biopolymer-based films. Layer-by-layer (LbL) assembly enables the sequential deposition of oppositely charged polymers such as chitosan or gelatin, forming nanostructured multilayered films with precisely controlled thickness (typically 10–500 nm per bilayer) and high functional tunability. These films are particularly attractive for applications requiring strong barrier, antioxidant or antimicrobial properties without bulk material modification [208,209,210].

3D printing (additive manufacturing) has also gained attention as it allows customized geometries, spatial distribution of active agents and real-time modulation of formulation architecture [211]. Meanwhile, blow-film extrusion remains the dominant industrial method for producing flexible biopolymer films with optimized mechanical and barrier properties [212,213,214].

These emerging approaches provide new opportunities to overcome limitations associated with traditional casting and coating methods, bridging laboratory-scale developments and industrial packaging solutions for cosmetic applications.

## 4. Fundamental Methodologies for Characterizing Active Packaging

**Figure 5:**
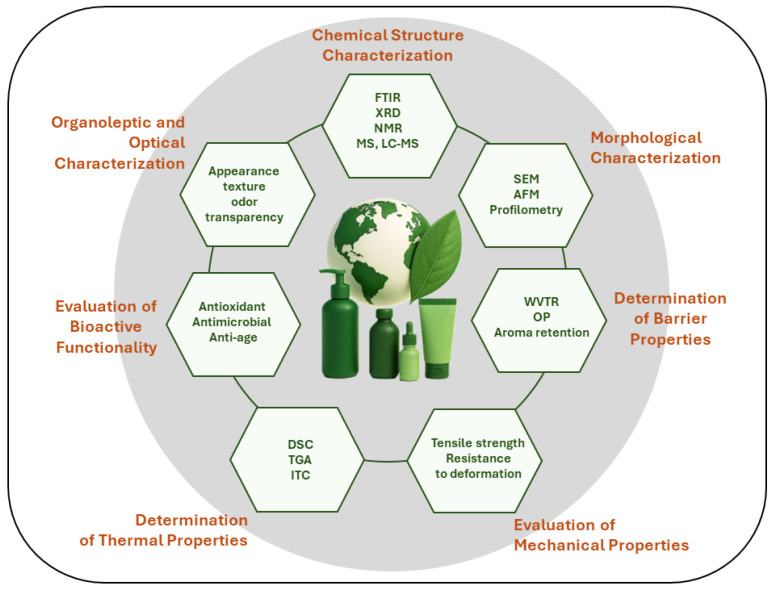
Overview of key analytical domains for the characterization of biomaterials in active cosmetic packaging. Each segment highlights the most common, essential, and mandatory techniques used to evaluate structural, functional, sensory, and environmental performance.

The development of active packaging systems for cosmetic applications demands a rigorous and multidimensional characterization approach. This involves the systematic application of chemical, morphological, mechanical, thermal, barrier, bioactive, optical, and sensory analyses, each supported by well-established and mandatory techniques. These methodologies are essential not only for ensuring material performance and regulatory compliance, but also for guiding formulation compatibility, user experience, and environmental alignment. Figure 5 illustrates the principal analytical domains and core techniques most applied in the characterization of biomaterials for active cosmetic packaging.

### 4.1. Chemical Structure Characterization

Chemical structure characterization is a fundamental step in the development of biopolymeric materials, as it provides insight into molecular architecture, functional group interactions, and compatibility between components. However, in the context of biopolymer-based active packaging, this characterization is not always explicitly reported. This omission often stems from the use of well-established materials—such as chitosan, gelatin, zein, kafirin and starch—whose chemical structures, degradation pathways, and biocompatibility profiles have been extensively studied and validated across multiple applications [2,100,102,108,215]. These polymers are frequently selected based on their known film-forming ability, biodegradability, and intrinsic bioactivity, which reduces the perceived need for repeated structural analysis. Nevertheless, when these matrices are functionalized with natural extracts, nanoparticles, or blended with other polymers, chemical characterization becomes essential to confirm molecular interactions, assess compatibility, and correlate structural modifications with performance attributes such as antioxidant capacity, antimicrobial efficacy, and barrier behavior [216,217,218]. Importantly, in cosmetic packaging applications, these interactions must be interpreted considering specific environmental and formulation-related stressors, including moderate temperatures (20–40 °C), fluctuating humidity (40–90% RH), variable pH (typically 4.5–7.5 for skin-compatible products), and prolonged exposure to aqueous, oily or emulsion-based matrices.

In this context, Fourier Transform Infrared Spectroscopy (FTIR) remains one of the most extensively employed techniques due to its accessibility and high diagnostic value. This technique is based on the absorption of infrared radiation by molecular bonds, which induces vibrational transitions specific to functional groups [219]. FTIR enables the identification of chemical moieties and interaction patterns, making it indispensable for confirming the incorporation of bioactive and monitoring polymer modifications. Recent studies have demonstrated its value in detecting hydrogen bonding and esterification in chitosan–gelatin films functionalized with flavonoids, where spectral shifts in the O–H and C=O regions correlate with enhanced antioxidant activity and mechanical strength [220]. These interactions are particularly relevant for packaging of aqueous and emulsion-based cosmetics, where hydrogen-bond networks strongly influence moisture uptake and structural integrity.

Similarly, X-ray diffraction (XRD) is another widely used technique which is used to evaluate the crystallinity and behavior of various biomaterials in different matrix systems. This method is based on the constructive interference of monochromatic X-rays scattered by atoms in a crystalline lattice, producing a diffraction pattern that reveals the degree of order, interplanar spacing, and phase composition [221]. In cosmetic-packaging systems, crystallinity plays a decisive role in controlling permeability, mechanical strength and long-term dimensional stability, especially in high-humidity environments such as bathrooms or humid climates. For example, Sun et al. [222] reported that the incorporation of brassica-derived compounds into chitosan–gelatin films disrupted crystalline packing, increasing amorphous content and improving flexibility—an advantageous feature for packaging of squeezable creams, gels and lotions. In contrast, the addition of layered double hydroxides (LDHs) promoted semi-crystalline domains, enhancing tensile strength and reducing permeability [223], features that are particularly beneficial for protective packaging of volatile or oxidation-sensitive oily formulations. These findings underscore XRD’s relevance in tailoring packaging properties through structural modulation [224].

In contrast, nuclear magnetic resonance (NMR) spectroscopy, although a powerful technique for structural elucidation, is less frequently used in this type of characterization studies. This technique is based on the magnetic properties of atomic nuclei—typically hydrogen (^1^H) or carbon (^13^C)—which resonate at characteristic frequencies when placed in a magnetic field and exposed to radiofrequency pulses [225]. The resulting spectra provides detailed information about the chemical environment, molecular dynamics, and structural conformation. In cosmetic-related applications, NMR has proven particularly valuable in confirming the chemical integrity of encapsulated bioactives and in elucidating interactions between polymers and hydrophobic or amphiphilic compounds commonly found in oil-based cosmetics [226,227]. Moreover, advanced solid-state NMR techniques have enabled the study of nano crystallization effects in cellulose-based films, revealing changes in mobility and segmental orientation that influence barrier performance [228]. Wang et al. [229] further emphasized the role of 2D NMR in resolving complex polysaccharide structures relevant to active packaging matrices.

On the other hand, Mass Spectrometry (MS), particularly when coupled with chromatographic separation and high-resolution tandem analysis (e.g., UHPLC-HRMS/MS), is increasingly used not only to quantify the migration of bioactive compounds from packaging films into cosmetic matrices, but also to monitor their structural integrity and possible transformation during release [230]. This technique, based on the ionization of chemical species and measurement of their mass-to-charge ratio (m/z), allows the detailed identification of low-molecular-weight compounds, characteristic fragmentation patterns, degradation products, and structural modifications. Recent studies employing UHPLC-HRMS/MS have demonstrated its capability to assess the preservation of phenolic and antioxidant compounds even under extreme conditions, confirming its robustness for tracking compound stability and transformation [230]. In cosmetic-packaging research, MS has been successfully applied to monitor the controlled release of antioxidants, preservatives or fragrances from biopolymer films into aqueous creams or lipid-rich matrices. For instance, Otero-Pazos et al. [231] demonstrated the application of MS to monitor the controlled release of tocopherol from chitosan-based films, highlighting its relevance for oxidative-stability preservation in oil-containing formulations such as serums and ointments.

Finally, Elemental Analysis [232], which consists of quantifying the elemental composition of a sample—typically carbon, hydrogen, nitrogen, and other relevant atoms—complements these techniques by confirming the presence of inorganic additives and assessing purity. This method is usually performed via combustion followed by detection of evolved gases, or through spectroscopic techniques such as atomic absorption or inductively coupled plasma optical emission spectroscopy (ICP-OES). In the context of cosmetic-packaging systems, elemental analysis is particularly useful for confirming the incorporation of metal oxides (e.g., ZnO, TiO_2_, Ag or Cu nanoparticles) and for determining the degree of deacetylation in chitosan, a key parameter directly related to antimicrobial efficacy and pH-dependent solubility in water-rich formulations (such as toners, gels and hydrophilic creams) [233].

Altogether, these techniques provide a robust analytical framework for the development of next-generation active packaging materials. Their integration allows researchers to correlate chemical structure with macroscopic performance under conditions that simulate real cosmetic storage and use, including moderate heat, high humidity, repeated contact with water or oils, and exposure to active ingredients. As such, the results obtained from these characterization techniques can be considered highly transferable to packaging applications for aqueous and emulsified products (e.g., creams, lotions, serums, masks) as well as lipid-rich or anhydrous formulations (e.g., balms, oils, solid cosmetics), provided that environmental conditions are considered during experimental evaluation.

### 4.2. Morphological Characterization

Morphological characterization has been established as a critical analytical dimension in the development of biopolymer-based active packaging systems, as surface features such as fiber diameter, roughness, and porosity directly influence barrier performance, mechanical cohesion, and the release behavior of bioactive compounds [234,235]. These structural attributes are particularly relevant for cosmetic packaging, where materials are exposed to repeated mechanical stress, variable humidity, and continuous contact with chemically diverse formulations.

Depending on the processing technique, biopolymer-based materials can exhibit markedly different morphological features. Electrospun matrices typically exhibit high surface area and interconnected porosity, cast and thermo-compressed films tend to form smoother, denser structures. While high porosity may enhance diffusion and active release, denser morphologies tend to provide superior mechanical resistance and barrier properties—features that are critical in cosmetic containers, sachets, liners and biodegradable wraps exposed to aqueous, oily or emulsified products.

To assess these features, scanning electron microscopy (SEM) has been widely employed; this technique operates by scanning a focused beam of electrons across the sample surface, generating secondary electrons that are detected to form high-resolution images of topography and microstructure [236,237]. SEM analyses performed on protein- and polysaccharide-based systems have provided valuable insights under conditions relevant to cosmetic packaging. For example, SEM analysis of SF/PCL nanofibers revealed that fiber diameter and distribution were modulated by processing parameters, directly affecting wettability and biocompatibility [238]. In starch-chitosan films loaded with *Gongju* extract, SEM provided critical insight into the surface morphology of films, revealing smoother and more homogeneous structures compared to control films [239]. In zein-based bilayer films, SEM revealed improved interfacial compatibility and surface uniformity when PEG400 was incorporated, resulting in enhanced mechanical strength and reduced water vapor permeability [240], an advantageous property for packaging of hydrophilic creams, masks and gel-based formulations.

In contrast, atomic force microscopy (AFM) has enabled nanometric topographic analysis based on the interaction between a sharp probe and the sample surface; the probe, mounted on a cantilever, deflects in response to surface forces, and these deflections are recorded to reconstruct three-dimensional maps of roughness and texture [241,242]. In cosmetic-related applications, AFM has proven particularly useful in correlating nanoscale roughness with functional activity. Increased surface irregularity has been linked to improved antimicrobial performance in thyme oil-enriched films, where improved contact between microorganisms and active surfaces was observed [243]. In fish gelatin films enriched with Aloe vera, AFM and FTIR revealed structural rearrangements and increased hydrophilicity due to polysaccharide–protein interactions, which influenced both roughness and thermal stability [244]. These effects are critical in packaging systems intended for aqueous cosmetics, where hydrophilicity and interfacial stability govern moisture absorption and structural integrity. Furthermore, zein films plasticized with polyols such as sorbitol and glycerol showed distinct AFM topographies, where sorbitol improved surface smoothness and reduced oxygen permeability [245], relevant to oxidation-sensitive products such as facial oils and antioxidant-rich serums.

On the other hand, profilometry has complemented these techniques by providing precise measurements of film thickness and continuity. Optical profilometry operates by projecting light onto the surface and analyzing reflected patterns to determine height variations, while mechanical profilometry uses a stylus that physically traces the surface profile [246,247]. These measurements are particularly important when designing cosmetic packaging materials, as thickness homogeneity minimizes weak points that can lead to rupture, deformation or uncontrolled migration [248,249]. In chitosan-based systems, thickness uniformity has been correlated with reduced water vapor transmission rates (WVTR), suggesting that microstructural consistency enhances barrier function in humid environments [250,251,252]. Kafirin films plasticized with triacetin and PEG400 exhibited improved surface homogeneity and reduced microporosity, as confirmed by profilometry and SEM, contributing to enhanced mechanical and barrier properties [253]. Starch-based films modified with nano clays and crosslinkers have also shown improved surface continuity and reduced roughness, as revealed by profilometric and SEM analyses [254], supporting their suitability for semi-rigid cosmetic packaging and protective inner linings.

Although these techniques differ in resolution, operational context, and sample-preparation requirements, their integration with chemical, thermal, and functional analyses has proven essential for a comprehensive understanding of biopolymer-based packaging systems. SEM combined with FTIR has revealed that porosity often arises from underlying molecular interactions, while AFM-derived roughness parameters have been linked to antimicrobial efficacy, and profilometry has provided quantitative support for barrier performance models. Beyond offering purely descriptive information, these morphological characterization techniques enable the rational design of packaging systems tailored to the preservation, aesthetic, and functional demands of cosmetic formulations.

In the specific context of cosmetic packaging, such morphological evaluations are especially relevant under conditions of high relative humidity (60–90% RH), skin-contact temperature ranges (25–40 °C), and repeated exposure to aqueous or lipid-based products. Surface defects, microcracks, and interconnected porosity detected by SEM, AFM, and profilometry directly correlate with increased moisture uptake, higher migration risk, and compromised microbiological protection in creams, gels, serums, and emulsions [255,256,257,258,259]. Therefore, the morphological stability observed in these biopolymer films can be considered highly representative of the performance expected in real cosmetic packaging environments, strengthening the translational relevance of the reported analyses.

### 4.3. Determination of Barrier Properties

Barrier properties have been recognized as essential performance indicators in the design of active packaging systems, particularly those intended for cosmetic formulations sensitive to moisture, oxygen, and volatile compound loss [260]. These properties determine the material’s ability to regulate mass transfer phenomena, including water vapor transmission rate (WVTR) [261], oxygen permeability (OP) [262], and aroma retention [263], which directly affect product stability, shelf life, and sensory integrity. While conventional petroleum-based polymers exhibit low permeability and high sealing efficiency, biopolymer-based films often require structural and compositional optimization to achieve comparable barrier performance [264,265,266].

In cosmetic applications, where formulations are stored for extended periods in humid environments and frequently exposed to air, light and temperature fluctuations, these barrier parameters become critical for preventing oxidation, dehydration, phase separation and loss of fragrance. Consequently, the evaluation of barrier properties under controlled but realistic conditions is essential to determine the practical feasibility of biopolymer-based packaging in the cosmetic sector.

To evaluate WVTR, gravimetric methods are commonly employed, wherein the film is sealed over a desiccant-containing chamber and exposed to controlled humidity; the rate of water uptake is then measured over time. This approach, standardized by ASTM E96 [267], allows quantification of moisture diffusion through the film matrix. In chitosan composite films, WVTR has been shown to decrease with increasing polymer concentration and crosslinking density, indicating that molecular packing and hydrogen bonding restrict water migration [250]. In fish gelatin films layered with PLA and PBAT, water vapor permeability was significantly reduced due to the hydrophobic nature of the outer layers and the lamination strategy, which created tortuous diffusion paths and improved thermal stability [268]. Additionally, fish gelatin films enriched with Aloe vera demonstrated moderate oxygen and water vapor barrier properties, while FTIR analysis revealed increased hydrophilicity due to polysaccharide–protein interactions [244]. These characteristics are particularly relevant for packaging of aqueous and semi-solid cosmetic systems such as gels, toners, masks and hydrophilic creams, where controlled moisture exchange is necessary to prevent excessive dehydration or microbial contamination.

Similarly, oxygen permeability is typically assessed using coulometric or manometric techniques, which measure the rate of oxygen transmission across the film under defined partial pressures. These methods reveal that the incorporation of polyphenolic compounds or essential oils can either enhance or impair barrier function depending on their miscibility and distribution within the matrix [220]. Zein-based bilayer films incorporating PEG400, and chitosan have shown improved water vapor barrier performance and near-complete UV protection, with WVTR values as low as 6.60 × 10^−11^ g·m^−2^·s^−1^·Pa^−1^, attributed to enhanced interfacial compatibility and reduced porosity [240]. These properties are particularly advantageous for antioxidant-rich cosmetic systems, botanical extracts and photo-sensitive formulations such as facial serums and vitamin-enriched creams.

Correspondingly, kafirin films plasticized with triacetin and PEG400 exhibited reduced water permeability and increased contact angle values [253], indicating an enhanced resistance to moisture penetration and oxygen diffusion, making such films particularly suitable for lipid-rich, anhydrous or oil-dominant cosmetic formulations, including balms, facial oils, solid perfumes, and ointments that are highly susceptible to oxidative degradation.

In contrast, aroma retention and volatile compound migration are often evaluated using gas chromatography coupled with headspace analysis, allowing precise quantification of active compound loss over time. Films enriched with thyme essential oil demonstrated improved aroma retention when formulated as multilayer structures, suggesting that diffusion pathways can be modulated by architectural design [243]. Starch-based films modified with montmorillonite clays and polycaprolactone showed reduced WVTR and OP due to improved matrix cohesion and tortuous diffusion paths, making them suitable for active packaging applications [269]. Moreover, permeability data must be interpreted in conjunction with morphological and mechanical analyses, as surface defects, porosity, and film thickness significantly influence barrier behavior. SEM and profilometry have revealed that microcracks and uneven deposition correlate with elevated WVTR and OP values, underscoring the need for structural uniformity [238]. Although barrier performance is often compromised in pure biopolymer films due to their hydrophilic nature, recent advances in composite and multilayer strategies have enabled significant improvements. For example, zein films blended with anthocyanins not only improved antioxidant activity but also enhanced barrier properties and enabled intelligent spoilage detection through colorimetric response [270]. These enhancements are attributed to tortuous diffusion paths and interfacial interactions that hinder molecular transport. Finally, barrier characterization provides critical insight into the functional viability of packaging systems, guiding formulation decisions and enabling the rational design of materials that preserve cosmetic integrity under diverse environmental conditions.

From a cosmetic packaging perspective, WVTR, oxygen permeability and aroma retention are especially critical for emulsions, oil-rich formulations, natural extracts and volatile-active products, which are highly sensitive to oxidation and moisture exchange. Most of the reported measurements are performed at 25–38 °C and 50–90% RH, moisture ranges that directly mimic storage and bathroom conditions [271,272]. Consequently, the barrier results reported for chitosan-, fish gelatin-, zein- and kafirin-based films are fully applicable to cosmetic formulations in aqueous, semi-solid and oily matrices, particularly for products with high antioxidant sensitivity.

### 4.4. Evaluation of Mechanical Properties

Mechanical properties are fundamental to the structural integrity and functional viability of active packaging systems, particularly those intended for cosmetic applications where flexibility, tensile strength, and resistance to deformation are critical [273,274]. These attributes determine the material’s ability to withstand handling, sealing, and environmental stress without compromising barrier performance or aesthetic quality [275]. While synthetic polymers typically offer superior mechanical strength, biopolymer-based films require careful formulation and processing to achieve comparable performance. Tensile strength (TS), elongation at break (EAB), and Young’s modulus are commonly used to evaluate mechanical behavior, typically measured using universal testing machines under controlled humidity and temperature, following standards such as ASTM D882, where the tensile and deformation tests are performed at 23 °C and moderate relative humidity (50% RH) [276], which closely approximate common cosmetic-use and storage conditions, including those found in indoor environments, bathrooms and during skin contact.

In chitosan–gelatin films, TS has been shown to increase with crosslinking and incorporation of polyphenolic compounds, which enhance intermolecular interactions and reduce brittleness [220]; moreover, SEM and profilometry have revealed that smoother and more homogeneous surfaces correlate with improved mechanical resilience, while rough or cracked films tend to fail prematurely under tensile stress [238]. These results are especially relevant for packaging of aqueous and semi-solid cosmetics (e.g., creams, gels, masks and lotions) that require materials with sufficient mechanical stability to prevent rupture or deformation when exposed to moisture and physical pressure.

In a similar vein, fish gelatin films layered with PLA and PBAT exhibited improved tensile strength and flexibility, attributed to the lamination strategy and interfacial adhesion between hydrophilic and hydrophobic layers [268]. This mechanical reinforcement is highly advantageous for flexible cosmetic packaging formats such as sachets, sachet-like containers, biodegradable wraps and inserts, where repeated bending and compression are frequent. In contrast, films enriched with Aloe vera demonstrated increased EAB and reduced stiffness, indicating enhanced plasticity due to polysaccharide–protein interactions [244]. Such characteristics are particularly suitable for soft, conformable packaging used for dermal-contact products such as masks, under-eye patches and flexible liners.

In prolamin-based systems, zein films plasticized with glycerol or sorbitol showed distinct mechanical profiles, where sorbitol improved tensile strength and reduced brittleness, while glycerol enhanced flexibility but lowered TS values [245]. These tunable mechanical responses are valuable in the context of oil-rich or antioxidant-based cosmetic formulations, including facial oils, serums and lipid-rich creams, which require packaging systems that combine moderate rigidity with sufficient flexibility to prevent cracking.

By contrast, kafirin films plasticized with triacetin and PEG400 exhibited higher TS and lower EAB compared to zein, attributed to stronger hydrophobic interactions and disulfide cross-linking within the protein matrix [253]. These features make kafirin-based films particularly suitable for rigid or semi-rigid applications such as compact cases, protective layers for powdered cosmetics, and inner liners for dry formulations, where higher stiffness and compressive resistance are required.

Finally, starch-based films modified with montmorillonite clays and crosslinkers demonstrated enhanced tensile strength and reduced water sensitivity, as the nanofillers improved matrix cohesion and stress distribution [240]. These mechanical advantages are relevant for secondary cosmetic packaging and protective outer layers, especially when products are exposed to fluctuating humidity conditions during transport and storage.

From a cosmetic-packaging perspective, mechanical resistance is particularly critical for squeeze-based containers, soft tubes, sachets, wraps, liners and inserts that undergo repeated deformation, friction and environmental stress [277]. Furthermore, cosmetic formulations typically contain water, oils, alcohols, surfactants and bioactive ingredients, all of which can interact with packaging materials and influence their mechanical stability over time. For this reason, mechanical tests conducted under moderate humidity and near-ambient or skin-contact temperatures can be considered highly representative for predicting real-use performance in cosmetic packaging systems [278].

Taken together, these findings indicate that the improvements in tensile strength, elongation, flexibility, and mechanical endurance observed in chitosan-, fish gelatin-, zein-, and kafirin-based films under experimentally controlled conditions are directly transferable to cosmetic packaging applications. Accordingly, their mechanical performance must be evaluated in conjunction with morphological and barrier properties, since formulation parameters—such as polymer ratios, plasticizer type, and processing methods—can be deliberately modulated to achieve materials that satisfy the mechanical requirements of cosmetic packaging while preserving biodegradability and functional integrity.

### 4.5. Determination of Thermal Properties

Thermal properties are among the most widely applied analytical parameters in the characterization of biopolymer-based packaging systems, not only because they reflect the material’s behavior under heat stress, but also due to their ability to generate rapid and reproducible thermal profiles that serve as molecular “fingerprints” [279,280]. These thermal signatures allow differentiation between biomaterials and even between variants of the same polymer, revealing subtle differences in molecular weight, residual solvents, and impurities derived from extraction, purification, or manufacturing processes [281,282,283]. Moreover, thermal analysis offers critical advantages in compatibility studies, where transitions such as glass transition (Tg) or melting point (Tm) can indicate miscibility or phase separation, and in decomposition studies, where onset degradation temperatures and mass loss profiles inform formulation stability and shelf-life.

In the context of cosmetic packaging, thermal characterization is especially important because materials are routinely exposed to moderate heat, fluctuating environmental conditions and prolonged contact with complex formulations containing water, oils, surfactants, alcohols, antioxidants and fragrances. Therefore, the interpretation of thermal transitions in biopolymer films must be directly linked to realistic storage, processing and usage conditions.

Differential scanning calorimetry (DSC) [284], it measures the heat flux required to increase the temperature of a sample relative to a reference, allowing for the detection of transitions such as Tg, Tm, and crystallization. In this way, DSC compares the energy absorbed or released during thermal events, which can be considerably improved through the modulation effect (modulated DSC) [285,286], significantly increasing resolution by superimposing sinusoidal temperatures to separate reversible from irreversible transitions. In contrast, thermogravimetric analysis (TGA) complements this characterization by measuring mass loss as a function of temperature, providing data on thermal degradation, moisture content, and compositional stability. When integrated, DSC–TGA offers a comprehensive view of both energetic and mass-related changes [287], which is particularly valuable for predicting material performance under cosmetic storage and handling conditions.

Less commonly, isothermal titration calorimetry (ITC) [288] has been used to study binding interactions and enthalpic compatibility between biopolymers and active ingredients, although its application in packaging remains exploratory. Nonetheless, in cosmetic contexts, this technique may offer valuable insight into polymer–bioactive affinity, particularly for antioxidant, phenolic, and essential-oil components incorporated within active packaging matrices.

In chitosan-based films, thermal stability has been shown to improve upon incorporation of flavonoids and crosslinkers, which enhance molecular cohesion and reduce chain mobility; with respect to chitosan-based systems, TGA analyses revealed that chitosan–gelatin matrices loaded with flavonoids exhibited increased onset degradation temperatures and reduced decomposition rates [220], indicating enhanced thermal resistance suitable for cosmetic packaging cosmetic packaging exposed to moderate heat and long-term storage. These improvements are especially relevant for aqueous and semi-solid cosmetic products such as creams, gels and lotions, which require packaging stability under bathroom and transportation conditions.

In the case of fish gelatin films integrated with tara gum, thermal stability was significantly enhanced when the gum was pre-treated by ball milling, as revealed by TGA and DSC analyses; this modification increased the onset degradation temperature and reduced the enthalpy of transition, suggesting tighter polymer packing and reduced chain mobility [289]. Similarly, fish gelatin films blended with alginate dialdehyde and pomelo peel–derived carbon dots (PCDs) exhibited concentration-dependent thermal behavior: low PCD contents enhanced stability through hydrogen bonding interactions, whereas higher concentrations promoted accelerated thermal decomposition [290]. Both these results demonstrate the feasibility of fine-tuning thermal stability for the packaging of hydrated or water-rich cosmetic systems, including masks, serums and hydrogels.

On the other hand, Zein-based films have demonstrated excellent thermal resistance due to their high content of hydrophobic amino acids and compact molecular structure; when blended with PEG400 and cast as bilayer films with chitosan and PVA, zein exhibited a stable thermal profile with minimal mass loss below 200 °C, and the incorporation of anthocyanins further improved antioxidant activity and thermal resilience [240], while DSC and TGA analyses of zein films plasticized with oleic acid revealed a reduction in Tg, indicating enhanced flexibility and oxidative stability [291]. These characteristics are particularly advantageous for oil-rich and antioxidant-sensitive cosmetic formulations such as facial oils, vitamin-enriched serums and botanical extracts.

Kafirin-based films plasticized with triacetin and PEG400 showed a reduction in Tg and an increase in thermal degradation temperature, as confirmed by DSC and TGA; this behavior was attributed to enhanced chain flexibility and reduced crystallinity [253], making them suitable for heat-sealable and semi-rigid cosmetic packaging formats, including inner linings and protective layers. Additionally, kafirin–polycaprolactone blends exhibited dual-phase degradation profiles, with initial mass loss attributed to PCL and delayed decomposition of kafirin [292], indicating potential for thermally stratified, multilayer cosmetic packaging systems.

Furthermore, it is worth highlighting the starch-based films reinforced with micro cellulose fibers that showed improved thermal stability at low load levels (1 wt%), as the fibers enhanced interfacial interactions and delayed thermal decomposition; however, higher fiber content disrupted crystallinity and reduced degradation temperature, highlighting the importance of controlled reinforcement strategies [293], while starch–chitosan blends crosslinked with citric acid showed increased Tg and reduced mass loss in TGA, indicating improved thermal integrity and reduced plasticizer volatility [294]. These thermally enhanced systems are particularly relevant for dry or powdered cosmetic products, where dimensional stability under warm conditions is essential.

Overall, thermal characterization provides a multidimensional understanding of biopolymer behavior under heat stress, enabling rational design of packaging systems that maintain integrity during processing and storage, while supporting compatibility with active cosmetic ingredients and functional additives.

From a cosmetic perspective, thermal stability is a critical parameter not only for product storage, but also throughout processing, filling and distribution. Cosmetic packaging is typically exposed to temperatures of approximately 20–25 °C under standard storage conditions, increasing to 30–45 °C during transportation, warehousing and use in warm or tropical environments [295]. In addition, packages are frequently subjected to microclimatic conditions approaching skin surface temperatures (∼32–37 °C), as well as to highly humid environments (60–90% RH) commonly found in bathrooms and poorly ventilated spaces [296,297,298]. This combined thermal and hygrometric stress can significantly affect the structural integrity, barrier performance and overall functionality of biodegradable and biopolymer-based packaging materials. Consequently, the thermal behavior reported for these films provides a realistic and reliable prediction of their performance under cosmetic handling, logistics and shelf-life conditions, including formulations containing essential oils, antioxidants and vitamins.

### 4.6. Evaluation of Bioactive Functionality

Bioactive functionality has emerged as a defining attribute in the development of active packaging systems for cosmetic applications, as it enables the controlled release or sustained presence of compounds with antioxidant, antimicrobial, anti-age, or regenerative properties [299]. These functionalities are typically achieved through the incorporation of natural extracts, essential oils, polyphenols, peptides, or metallic nanoparticles into biopolymer matrices, which act not only as carriers but also as modulators of release kinetics and stability [300,301,302]. Depending on the formulation strategy, bioactive agents may be physically entrapped, chemically bonded, or dispersed as micro- or nano-sized inclusions, influencing their diffusion, interaction with the substrate, and long-term efficacy.

In the context of cosmetic applications, this functionality is especially relevant because packaging materials are in continuous or intermittent contact with water-rich, oil-rich or amphiphilic formulations, often at mildly acidic pH values (4.5–6.5) and under humid conditions (60–90% RH), which can both accelerate degradation processes and promote microbial growth [303,304,305]. Therefore, evaluating bioactive functionality under such conditions is fundamental in determining the real-world applicability of biopolymer-based active packaging systems in the cosmetic sector.

Chitosan-based films, their bioactivity has been extensively documented due to the intrinsic antimicrobial properties of chitosan and its ability to form electrostatic complexes with negatively charged microbial membranes. When combined with flavonoids or essential oils, chitosan matrices have demonstrated synergistic effects, enhancing both antioxidant capacity and microbial inhibition. For instance, chitosan–gelatin films loaded with flavonoids exhibited sustained antibacterial activity against *Staphylococcus aureus* and *Escherichia coli*, while also protecting sensitive cosmetic ingredients from oxidative degradation [220]. These properties are directly relevant for packaging of aqueous and semi-solid cosmetic products—such as creams, gels, masks and toners—where high water activity favors microbial proliferation and oxidative degradation.

In contrast, fish gelatin systems, bioactivity has been imparted through the incorporation of Aloe vera, green tea extract, or silver nanoparticles, which interact with the protein matrix to modulate release and enhance stability. Fish gelatin films enriched with Aloe vera gel showed improved antioxidant activity and microbial inhibition, attributed to the synergistic interaction between polysaccharides and bioactive phytochemicals [244]. Additionally, chitosan blends containing green and black tea extracts demonstrated prolonged radical scavenging activity, making them suitable for cosmetic preservation and topical application [306]. These effects are particularly relevant for hydrophilic cosmetics and emulsified systems, where antioxidant and antimicrobial protection must be sustained over extended shelf-life periods under humid conditions.

In the case of zein-based films, these have been used as carriers for hydrophobic bioactives, including curcumin, anthocyanins, and essential oils, due to zein’s amphiphilic nature and film-forming capacity. Zein–anthocyanin bilayer films exhibited pH-sensitive color changes and antioxidant activity, enabling intelligent spoilage detection and active protection in cosmetic formulations [270]. Moreover, zein–gum Arabic–tannic acid nanoparticles loaded with curcumin showed enhanced photostability and controlled release, supporting their use in UV-protective and anti-inflammatory cosmetic systems [307]. These features are highly advantageous for oil-rich, antioxidant-based and UV-sensitive cosmetic systems such as facial serums, botanical oils and anti-aging formulations.

While kafirin films, although less explored, have demonstrated promising bioactive potential when combined with plant-derived antimicrobials or metallic agents. Chitosan–silver nanocomposite films exhibited broad-spectrum antimicrobial activity and maintained structural integrity under cosmetic pH conditions, suggesting their suitability for preservative-free packaging formats [308]. In addition, kafirin-based films incorporating citral and quercetin demonstrated notable antimicrobial activity, attributed to the synergistic effects of the essential oil and polyphenol within the protein matrix. Such multifunctional behavior highlights their potential as active packaging materials for cosmetic formulations containing natural oils or labile bioactives, where preservation and protection against microbial contamination are essential [110]. This multifunctionality is especially suitable for anhydrous or oil-dominant cosmetics—such as balms, ointments and solid perfumes—where oxidation control and microbial protection are critical.

In addition, starch-based films have been functionalized with phenolic compounds, essential oils, and antimicrobial peptides to enhance their bioactivity. Starch–chitosan films loaded with cinnamon essential oil demonstrated strong antibacterial and antifungal activities [309]. Furthermore, thermoplastic starch films containing nisin and lysozyme showed synergistic antimicrobial effects and maintained bioactivity over extended storage periods, supporting their use in cosmetic preservation and hygiene applications [310]. These properties are particularly relevant for secondary cosmetic packaging and hygiene-related applications, including dry products and powder-based formulations, where environmental moisture must be controlled, and microbial contamination avoided.

Beyond these material-specific examples, recent studies have emphasized the importance of understanding the molecular mechanisms underlying bioactive functionality. Silver nanoparticles and chitosan–Ag composites exert antimicrobial effects by generating reactive oxygen species (ROS), binding to thiol groups in microbial proteins, and disrupting cell wall integrity [311,312]. Essential oils such as citral and terpenes destabilize microbial lipid bilayers, increasing membrane permeability and causing leakage of intracellular contents [313]. Proteinaceous antimicrobials like nisin and lysozyme complement these effects by forming pores in bacterial membranes and hydrolyzing peptidoglycan [314]. In contrast, phytochemicals such as catechins (from green and black tea extracts), curcumin, anthocyanins, quercetin, and Aloe vera extracts exhibit antioxidant activity through proton or electron donation, resonance stabilization of phenoxyl radicals, and chelation of transition metals (Fe^2+^, Cu^2+^), thereby preventing oxidative chain reactions [315,316,317]. Importantly, biocompatibility analyses using keratinocytes and other skin-relevant cell models confirm that these bioactive compounds, when applied at concentrations suitable for cosmetic packaging, maintain cellular viability and do not induce significant cytotoxicity, supporting their safe use in topical applications [312,314].

Overall, the incorporation of bioactive compounds into biopolymer-based films enables the development of multifunctional packaging systems that not only protect the physical and chemical integrity of cosmetic products but also contribute actively to microbial control, oxidative stability and skin safety. In cosmetic applications, bioactive functionality is especially critical for anti-aging creams, antioxidant serums, botanical emulsions and preservative-free products, which are highly susceptible to degradation and microbial contamination [318,319].

### 4.7. Organoleptic and Optical Characterization

Organoleptic and optical characterization plays a critical role in evaluating biopolymer-based packaging systems for cosmetic applications, as these properties directly influence consumer perception, product acceptance, and functional integration with the final formulation [320,321]. Organoleptic attributes—such as visual appearance, surface texture, tactile response, odor, and transparency—are essential not only for aesthetic compatibility but also for usability, especially in dermal and facial applications [322]. These parameters are typically assessed through a combination of instrumental techniques (colorimetry, gloss meters, profilometry, opacity analysis) and descriptive sensory panels, which provide qualitative and quantitative feedback on material performance under realistic conditions.

In the case of chitosan-based films, organoleptic properties have been modulated through blending and plasticization strategies; for example, the incorporation of glycerol or sorbitol has improved flexibility and reduced brittleness, while the addition of essential oils has altered odor and surface gloss. Chitosan–gelatin films loaded with flavonoids showed increased yellowness and reduced transparency, as measured by CIE Lab parameters, which correlated with antioxidant content and UV-blocking capacity [220]. These features are especially relevant for cosmetic creams, anti-aging products and photoprotective formulations, where partial opacity and UV-shielding properties are often desirable.

In fish gelatin-based systems, visual and tactile properties have been influenced by protein concentration, drying conditions, and additive incorporation; films enriched with Aloe vera gel demonstrated enhanced smoothness and reduced surface irregularities, as confirmed by profilometry and sensory panel evaluation, while maintaining neutral odor and high transparency [244]. Similarly, fish gelatin/alginate blend exhibited improved mechanical properties (greater elongation and higher tensile strength) and good compatibility, suggesting a more homogeneous matrix [323].

Regarding zein-based films, their natural golden hue and hydrophobic surface have been modulated through pH adjustment and plasticizer selection; zein–anthocyanin bilayer films exhibited pH-responsive color shifts and reduced gloss, enabling intelligent packaging with visual spoilage indicators [270]. This property opens opportunities for intelligent cosmetic packaging capable of indicating product degradation, oxidation or contamination. Additionally, zein films plasticized with oleic acid showed increased surface smoothness and reduced opacity [324], enhancing their suitability for transparent or translucent packaging formats such as cosmetic wraps, sachets and inner liners. These characteristics are especially relevant for oil-based or antioxidant-rich formulations, where both visual clarity and protective function are required.

In addition, kafirin-based films exhibit matte finishes and neutral tones that can be tuned through extraction and processing conditions. Films cast from lyophilized kafirin extracted with NaOH demonstrated superior sensory attributes—showing greater clarity, lighter color, enhanced flexibility, and smoother surface texture—compared to those prepared from non-alkali-extracted kafirin dried at 40 °C, which appeared darker, less transparent, and markedly rougher to the touch [325]. These tunable aesthetic features support their potential application in both minimalist and natural-style cosmetic packaging, particularly for solid or semi-solid products such as compact powders, solid perfumes and botanical balms.

From a cosmetic-packaging perspective, organoleptic and optical characteristics are also strongly affected by the nature of the contained formulation. Aqueous and hydrogel-based cosmetics require packaging with high transparency, low haze and neutral odor to preserve clarity and consumer trust [326]. Emulsions and semi-solid products (e.g., creams and lotions) benefit from slightly opaque, UV-protective materials that minimize light-induced degradation [327]. Oil-rich and anhydrous formulations require stable surface properties and low opacity changes to prevent oxidation and fragrance alteration, whereas dry and powder-based cosmetics demand materials with matte finish, low tackiness and high mechanical integrity to avoid stickiness, caking and product contamination [328,329].

Furthermore, exposure to high humidity levels (60–90% RH), moderate temperatures (25–40 °C), skin-contact conditions (32–37 °C), and mildly acidic environments (pH 4.5–6.5) can alter optical clarity, color stability, surface gloss and odor adsorption of biopolymer films [330,331]. Consequently, the fact that most reported organoleptic and optical analyses are conducted under conditions comparable to cosmetic environments reinforces the high transferability and validity of these results for real cosmetic packaging systems.

Overall, organoleptic and optical characterization provides not only aesthetic and sensory validation, but also valuable functional information linked to photostability, surface interactions, consumer perception and overall product quality. Therefore, these analyses represent a crucial component in the design of next-generation sustainable packaging materials tailored specifically for the complex physicochemical and sensory demands of modern cosmetic formulations.

Accordingly, and building upon the preceding analysis, Table 2 presents a structured synthesis of representative systems categorized by based biomaterial, incorporated compounds, reported functional properties, and potential cosmetic packaging application. This comparative framework highlights the technological relevance of each material–compound combination, emphasizing attributes such as mechanical enhancement, antioxidant and antimicrobial activity, controlled release, and biodegradability. The table also identifies indexed references that consolidate and substantiate these findings, offering a concise yet comprehensive overview of the functional potential of biopolymer-based systems for active cosmetic packaging.

## 5. Challenges and Future Perspectives: Transferability to the Cosmetics Sector

The implementation of biopolymer-based active packaging in the cosmetics industry presents a promising yet complex opportunity. While the food sector has demonstrated the efficacy of these systems in preserving product quality and extending shelf life, their transfer to cosmetic applications requires careful consideration of formulation compatibility, regulatory frameworks, consumer expectations, and industrial scalability.

One of the main advantages of these biopolymers—such as chitosan, fish gelatin, zein, and kafirin—is their biodegradability, renewable origin, and ability to form packaging systems composed of 100% natural ingredients, which agrees with current consumer trends favoring clean-label and eco-conscious products. These materials also enable the use of functional claims such as “antioxidant protection,” “microbial safety,” or “natural preservation,” which can enhance product differentiation and marketing appeal.

Several challenges must be addressed to ensure the successful integration of biopolymer-based active packaging into the cosmetics sector. First, cost and scalability remain critical limitations, as these systems are generally more expensive than conventional synthetic materials due to the sourcing, purification, and processing of natural raw materials. Second, sensory attributes such as odor and coloration—particularly in fish gelatin and plant-derived proteins—may affect consumer acceptance; however, these effects can be mitigated through deodorization, masking agents, or composite formulations. Third, natural biopolymers often exhibit lower mechanical strength, higher water vapor permeability, and reduced thermal stability compared to synthetic polymers, necessitating reinforcement strategies such as blending or multilayer structuring. Fourth, although many biopolymers meet food-grade safety standards, their application in cosmetics may require additional toxicological validation, especially for direct-contact packaging. Finally, formulation compatibility must be carefully considered, as cosmetic products vary widely in pH, viscosity, and chemical composition, and packaging materials must be tailored to avoid adverse interactions that compromise product integrity or stability.

From an economic perspective, the development of biopolymer-based active packaging for cosmetics currently faces significant cost-related challenges compared to petroleum-derived materials such as polyethylene, polypropylene, or PET. The production of natural polymers involves higher raw material and processing costs due to factors such as limited supply chains, purification requirements, and less mature industrial infrastructure. Moreover, the incorporation of bioactive compounds, nanoparticles, or essential oils to confer functional properties further increases formulation and manufacturing expenses. In contrast, synthetic plastics benefit from decades of industrial optimization, large-scale petrochemical production, and lower per-unit costs. Nevertheless, when considering the full life-cycle costs—including waste management, recyclability, and environmental taxation—biopolymer systems may offer long-term economic advantages aligned with circular economy models. As regulatory and consumer pressures drive the cosmetic industry toward sustainable packaging, advances in biopolymer processing, valorization of agro-industrial byproducts, and economies of scale are expected to progressively reduce production costs, narrowing the gap with conventional petroleum-based materials.

Despite these challenges, the feasibility of transferring active packaging systems to the cosmetics sector is high, particularly for formulations vulnerable to oxidation, microbial contamination, or light-induced degradation. Advances in processing technologies, composite film design, and functionalization strategies continue to expand the applicability of these materials, offering a sustainable and innovative alternative to conventional packaging.

## 6. Conclusions

This review highlights the growing relevance of biopolymer-based active packaging systems as sustainable and functional alternatives for the cosmetics industry, drawing on technological advances and validated applications from the food sector. Materials such as chitosan, fish gelatin, zein, and kafirin exhibit biodegradability, compatibility with natural additives, and intrinsic but limited antioxidant and antimicrobial properties, which can be significantly enhanced through the incorporation of bioactive compounds. These systems enable the development of packaging composed entirely of renewable ingredients and support clean-label claims in line with current consumer trends. Despite challenges related to cost, mechanical performance, sensory attributes, and regulatory adaptation, these biopolymers offer feasible solutions for protecting oxidation-prone and microbiologically sensitive cosmetic formulations. The integration of tailored processing techniques and composite strategies further enhances their applicability, positioning active packaging as a viable innovation pathway toward safer, more stable, and environmentally responsible cosmetic products. While these technologies have gained substantial traction in the food industry over the past decade, their adoption in cosmetics remains limited. This gap represents a strategic opportunity to redirect attention toward food-derived systems and adapt them to the specific needs of cosmetic formulations, especially in light of emerging ecological values and consumer expectations that increasingly favor sustainable, preservative-free, and naturally packaged cosmetic products.

## Figures and Tables

**Table 1:** Comparative analysis of biopolymers studied and their advantages/disadvantages for cosmetic packaging applications.

Biopolymer	Advantages	Disadvantages
Chitosan	Biodegradable; antimicrobial and antioxidant activity; compatible with plant extracts; film-forming; supports natural claims	Higher cost than synthetics; limited solubility at neutral pH; may require plasticizers or blending
Fish Gelatin	Renewable origin; good film-forming ability; compatible with bioactives; biodegradable; supports clean-label claims	Odor and low thermal/mechanical stability; sensitive to humidity; may require composite reinforcement
Zein	Hydrophobic; good barrier properties; thermal stability; antioxidant potential; food-grade; supports UV protection claims	Brittle without plasticizers; yellow coloration; limited solubility; moderate cost
Kafirin	Strong hydrophobicity; stable films; antioxidant and antimicrobial potential; compatible with essential oils	Difficult extraction; low solubility; residual lipids; limited industrial availability
Starch (complementary)	Abundant; biodegradable; low cost; compatible with essential oils and antioxidants; supports “natural origin” claims	Poor water resistance; low mechanical strength; requires crosslinking or reinforcement

**Table 2:** Representative biomaterial-based systems and incorporated compounds used in active cosmetic packaging. The table summarizes reported functional properties—including mechanical enhancement, antioxidant and antimicrobial activity, controlled release, and biodegradability—and outlines their potential applications in cosmetic formulations and packaging formats. References correspond to indexed studies supporting each material–compound combination.

Based Biomaterial	Functional Ingredient	Preparation Method	Reported Functional Properties	Potential Cosmetic Packaging Application	Ref.
Chitosan	Tea tree oil in modified palygorskite	Solvent casting method	Enhanced mechanical strength, fluid absorption, controlled release (10 h), antimicrobial activity (*P. acnes*, *S. aureus*), antioxidant activity (93% DPPH).	This composite film is suitable for solid or semi-solid cosmetic formats such as acne patches, solid bars, and therapeutic spot dressings, where its antimicrobial and antioxidant activities provide functional value. Due to the hydrophilic and swelling-prone nature of chitosan, direct long-term contact with aqueous emulsions or gels is not recommended. Instead, it can be incorporated as an antimicrobial liner or internal protective sheet within containers for natural or preservative-free formulations.	[332]
Chitosan	Naringenin, apigenin, luteolin	Solvent casting method	Improved strength, thermal stability, antioxidant activity (ABTS: luteolin > naringenin > apigenin), antibacterial activity (*S. aureus*).	The film is appropriate for dry or low-moisture cosmetics such as antioxidant facial patches, solid balms, and compact topical bars, where controlled release and phenolic bioactivity are advantageous. Its water sensitivity limits direct use with high-moisture emulsions, but it may act as an internal stabilizing insert to reduce oxidative degradation in natural creams or serums. This makes it particularly relevant for formulations requiring enhanced oxidative protection.	[220]
Chitosan/Zein	Zein/chitosan nanoparticles, tea polyphenols, cinnamaldehyde	Solvent casting method	Significant increase in antioxidant activity (57.6% DPPH), antibacterial activity (*S. aureus*, *E. coli*), improved structural stability.	Improved moisture tolerance due to zein incorporation enables application in semi-solid or moderately hydrated cosmetics, including balms, butters, or anhydrous creams. Its antimicrobial and antioxidant performance also supports use as an active inner coating for natural emulsions. While not optimal as primary packaging for water-rich systems, it provides functional stabilization and shelf-life extension as a barrier-enhancing internal layer.	[333]
Chitosan	Kaolin clay and biovanillin	Solvent casting method	Antioxidant activity (80% DPPH), antimicrobial activity (90% reduction of *E. coli* and *S. aureus*), antifungal activity (75% reduction), biodegradability (88% within 20 days).	This biodegradable film is suitable for solid cosmetic formats such as cleansing bars, dry masks, or pressed balms, where its antioxidant, antimicrobial, and antifungal effects enhance preservation. Its moisture sensitivity limits direct exposure to liquid formulations, but it can serve as an internal sachet or secondary liner to protect oxidation-prone creams. This makes it particularly useful for clean-label, low-preservative cosmetic systems.	[334]
Chitosan	Calcium oxide nanoparticles	Solvent casting method	Outstanding antimicrobial properties (>99.999% reduction of *S. aureus* and *E. coli*), improved thermal and mechanical resistance, reduced water vapor permeability.	The composite exhibits strong antimicrobial activity and enhanced barrier performance, making it suitable for solid or semi-solid cosmetics that require microbiological protection, such as deodorant bars or compact skincare sticks. It may be employed as an antimicrobial lid liner or insert for preservative-free creams. However, its hydrophilicity still restricts direct long-term contact with aqueous emulsions.	[31]
Chitosan	Cell-free supernatant (CFS) of *Lacticaseibacillus paracasei* ALAC-4	Solvent casting method	Strong antifungal activity against molds and yeasts (notably *Candida albicans*); improved mechanical strength; formation of hydrogen bonds between CS and CFS (FTIR evidence); smooth and compact morphology (SEM).	This antifungal film is appropriate for dry facial masks, compressed mask sheets, or powder-to-gel systems where fungal contamination is a risk. In aqueous or emulsion-based cosmetics, it may act as an internal antifungal protective layer, reducing mold and yeast growth without functioning as the primary contact surface. Its bio-based origin aligns well with natural and probiotic-inspired cosmetic lines.	[335]
Chitosan/Guar gum composite	Watermelon rind extract (WRE)	Solvent casting method	Formation of hydrogen-bonded intermolecular network (FTIR, XRD, SEM evidence); improved mechanical strength and barrier properties (reduced WVTR and O_2_ permeability); enhanced antioxidant activity (83.24% DPPH) and antibacterial effect; sustainable bio-based additive from agro-waste.	The film is suitable for low-moisture and solid cosmetic applications such as bars, balms, and powder masks, benefiting from its antioxidant and antimicrobial activities. Although not ideal as primary packaging for liquid formulations, it may serve as an internal protective insert for natural emulsions susceptible to oxidation. Its sustainability profile makes it particularly attractive for eco-conscious brands.	[336]
Chitosan	Scallion flower extract (SFE)	Solvent casting method	Enhanced mechanical and oxygen-barrier properties due to hydrogen bonding and electrostatic interactions; improved antioxidant activity (DPPH 74.8%, ABTS 84.1%) and antibacterial performance (1.9x vs. control); reduced water solubility and moisture content; eco-friendly (no toxic reagents).	This bioactive film is well-suited for solid or semi-solid cosmetic formats requiring antioxidant and antimicrobial enhancement, such as cleansing bars or solid serums. For creams and gels, it functions best as an internal barrier layer that reduces microbial and oxidative degradation. Its reduced solubility improves performance but does not fully overcome limitations in high-moisture environments.	[337]
Chitosan	Zinc oxide nanoparticles loaded with gallic acid (ZnO@gal)	Solvent casting method	Improved mechanical strength and elongation; reduced oxygen and water vapor permeability; decreased water solubility and swelling; strong UV–VIS light barrier; enhanced antioxidant and antibacterial activity; homogeneous nanoparticle dispersion (SEM); hydrogen bonding interactions between CS and ZnO@gal (FTIR).	The composite’s UV-protective and antimicrobial properties make it valuable for solid or oil-based cosmetics sensitive to light or oxidation, including balms, sticks, and facial oils. In emulsions or serums, it can act as a UV-protective internal liner to mitigate photodegradation. Its reduced water solubility improves stability but still limits direct-intimate contact with high-moisture matrices.	[338]
Poly(ε-caprolactone)/Chitosan (PCL/CS) nanofiber composite	Urchin-like gold nanoparticles (UGNP)	Electrospinning	Enhanced antibacterial activity against *S. aureus* and *E. coli* (dependent on UGNP spine length and loading); good thermal stability, hydrophilicity, and mechanical strength; optimized water vapor permeability; improved surface interaction due to topographic nanostructure.	This nanofibrous system provides antimicrobial and barrier performance suitable for high-value semi-solid cosmetics such as gel patches, sheet masks, or serum-infused pads. Its structural robustness supports use as an inner functional layer in containers for natural emulsions, though chitosan restricts use as primary packaging for water-rich systems. Engineered nanofiber morphology enhances interfacial stability and protective functionality.	[339]
Chitosan	ɛ-Polylysine (ɛ-PL)	Solvent casting method	Improved thickness and tensile strength; reduced water vapor permeability (WVP); enhanced antioxidant and antimicrobial properties (synergistic effect between CS and ɛ-PL); significant inhibition of microbial growth (total viable count, coliforms, molds, yeasts) and oxidative degradation; non-toxic and biodegradable.	This composite film is suitable for solid and semi-solid cosmetic formats requiring enhanced antimicrobial protection, such as natural deodorant bars, cleansing sticks, or solid balms. In emulsions or gels, it performs best as an internal antimicrobial insert that suppresses bacterial, yeast, and mold proliferation without being in continuous direct contact with water phases. Its synergistic antimicrobial activity is particularly valuable for preservative-free cosmetic systems.	[340]
Chitosan	Eugenol nanoemulsion (EuNE), Aloe vera gel (AVG), Zinc oxide nanoparticles (ZnONP)	Solvent casting method	Improved UV-barrier, antioxidant activity (up to 95% DPPH scavenging), antimicrobial activity (enhanced antibacterial performance), increased tensile strength, improved hydrophobicity, reduced solubility, maintained transparency, good component compatibility (XRD).	This multifunctional film is appropriate for solid or anhydrous cosmetic products, where its UV-protective, antioxidant, and antimicrobial performance benefits stability. In light-sensitive creams or serums, it can serve as an internal UV-shielding and antimicrobial liner, reducing degradation while avoiding direct immersion in aqueous matrices. Its enhanced hydrophobicity improves tolerance to moisture but still limits use as primary packaging for liquid formulations.	[341]
Chitosan	Luteolin	Solvent casting method	Improved microstructural compactness and homogeneity, enhanced water vapor and oxygen barrier, increased mechanical strength, controlled release of luteolin (up to 10 days), antioxidant and antimicrobial activity, high encapsulation efficiency (89.52%), zeta potential −39.8 mV.	This antioxidant and antimicrobial film is suitable for solid balms, solid serums, and pressed cosmetic formats where controlled release of luteolin enhances stability. For emulsions and serums, it functions effectively as an internal controlled-release antioxidant layer that delays oxidative deterioration. Its improved barrier properties offer added protection but do not fully overcome chitosan’s incompatibility with continuous water exposure.	[342]
Chitosan	Crude *Brassica* extract	Solvent casting method	Enhanced mechanical strength, light transmittance, and oxygen/water vapor barrier properties; improved antioxidant and antimicrobial activity due to *Brassica* extract; compatible and homogeneous chitosan/gelatin matrix confirmed by FTIR and SEM; overall active film performance superior to control formulations.	The composite film is ideal for solid and semi-solid cosmetics enriched with natural oils or botanical extracts, benefiting from its antioxidant and antimicrobial activity. In emulsions, it may be used as a secondary liner that enhances microbial and oxidative stability while minimizing direct contact with aqueous phases. Its structural integrity and bioactivity align well with eco-friendly and “clean beauty” packaging approaches.	[222]
Chitosan	Thyme essential oil (TEO)	Solution casting method	Enhanced mechanical strength (TS = 7.43 MPa), elongation at break (up to 28.22%), and barrier properties (improved WVP and OP); formation of hydrogen bonds between chitosan, pectin, and TEO confirmed by FTIR; significant antimicrobial activity (complete inhibition of *S. typhimurium* at 20% TEO); microstructural homogeneity improved by SEM and AFM analysis.	This antimicrobial active film is suitable for solid soaps, cleansing bars, and semisolid balms where fungal and bacterial contamination is a concern. It may be incorporated as an internal antimicrobial barrier in containers for natural creams or gels, providing surface protection without functioning as the primary moisture-exposed material. Its enhanced mechanical properties support usage in solid cosmetic applications.	[243]
Chitosan	Hydroxyapatite nanoparticles (HASP) derived from eggshell waste	Solvent casting method	Films with 3 wt% HANP showed 61.5% higher tensile strength and 1 wt% HANP film reduced WVP by 52%; improved thermal stability (higher Tg) and storage modulus; homogeneous nanoparticle dispersion confirmed by SEM/TEM/XRD; FTIR and XPS indicated strong Chitosan–HANP interactions.	The reinforced film is suitable for solid and semi-solid products that require strong barrier performance, such as facial bars, powdered cosmetics, and compact balms. In emulsions, it may serve as an internal oxygen- and moisture-reducing insert, extending stability without prolonged direct contact with the liquid phase. Its improved thermal and mechanical performance makes it an attractive biodegradable alternative to synthetic barrier materials.	[217]
Fish Gelatin	Ethanolic extract of *Lepidium sativum* seeds.	Solvent casting method	Preservation of thickness, tensile strength, and elongation, with slight alteration in color. High antioxidant capacity and reduction of bacterial growth.	This antioxidant and antimicrobial gelatin film is best suited for solid or oil-rich cosmetic formulations where its bioactivity enhances preservation. Due to its hydrophilicity, it is not ideal for direct contact with aqueous emulsions but may function as an internal protective liner that delays oxidation and microbial growth. Its biodegradability aligns well with sustainable cosmetic packaging initiatives.	[343]
Fish Gelatin	Pomegranate peel powder	Solvent casting method	Improvement in tensile strength and water vapor permeability. Enhanced antioxidant activity measured by DPPH and ABTS assays, with inhibition of *S. aureus*, *L. monocytogenes*, and *E. coli*.	This bioactive film is appropriate for solid cosmetics, makeup pans, and balm-based formulations that benefit from its antioxidant and antibacterial properties. In emulsions or creams, it can be used as an internal antioxidant layer enhancing shelf life while avoiding continuous water exposure. Its improved mechanical and barrier performance makes it suitable for eco-friendly packaging solutions.	[344]
Fish Gelatin	*Artemisia sphaerocephala* gum and bamboo flavonoids	Solvent casting method	Improvement in mechanical strength, barrier properties, and thermal stability, with reduction in elongation and permeability. Strong antioxidant and antimicrobial effects.	The mechanically reinforced and antioxidant-rich film is suitable for solid or semi-solid cosmetic formats, particularly those sensitive to oxidation or microbial contamination. As a liner for oil-based creams or anhydrous balms, it enhances stability while protecting bioactive compounds. Its limited water resistance still restricts direct contact with high-moisture emulsions.	[345]
Chitosan/Fish Gelatin	Chitosan and mango peel carbon dots	Solvent casting method	Improvement in mechanical strength, hydrophobicity, and UV barrier capacity, with additional luminescent properties. Outstanding antioxidant and antimicrobial effects.	This intelligent, luminescent, antimicrobial film is well-suited for solid cosmetics or semi-solid anhydrous formulations that benefit from visual detection and active protection. It can act as a UV-shielding and antioxidant liner inside packaging for serums or natural emulsions requiring enhanced stability. Due to hydrophilicity from both biopolymers, it should not be used as the primary packaging for liquid products.	[346]
Fish Gelatin	Essential oils (clove and oregano)	Extrusion method	Improvement in elasticity and elongation with a decrease in oxygen barrier properties. High antioxidant activity from clove and strong antimicrobial effects from oregano.	This active film provides strong antimicrobial and antioxidant protection, making it suitable for solid or oil-based cosmetics such as balms, makeup cakes, and solid perfumes. Its elasticity and bioactivity offer stabilization against microbial contamination in low-moisture formats. However, due to its high hydrophilicity, it is not recommended for direct-contact packaging of aqueous emulsions or gels but may be used as an internal antimicrobial liner.	[347]
Fish Gelatin	Liquid smoke (phenols and organic acids)	Solvent casting method	Improvement in water resistance and vapor barrier properties. Strong antimicrobial effect and inhibition of lipid oxidation.	Enhanced water resistance and vapor barrier properties allow this film to serve as protective packaging for semi-solid balms, ointments, or oil-based cosmetics. Its potent antimicrobial action makes it effective as an internal coating for containers storing natural emulsions, reducing lipid oxidation and surface contamination. Nonetheless, its limited moisture tolerance restricts use as direct-contact packaging for high-moisture formulations.	[348]
Fish Gelatin	Maillard reaction products (gelatin and fructose)	Solvent casting method	Improvement in mechanical strength, water vapor barrier properties, and reduction of light transmission. Strong antioxidant activity.	This thermally and mechanically improved film is suited for packaging oil-based cosmetics, facial oils, and solid balms that require antioxidant protection and reduced light-induced degradation. Its improved water vapor barrier enhances stability of oxidative-sensitive actives. Still, the hydrophilic nature of gelatin prevents safe direct use with aqueous formulations, making it more suitable as an inner liner rather than primary contact material.	[349]
Fish gelatin (from mackerel skin)	*Ficus carica L.* leaf extract (FLE)	Solvent casting method	Enhanced tensile strength (2.74 MPa), elongation at break (372.82%), reduced solubility (36.2%), low water vapor permeability (3.96 × 10^−11^ g/msPa), antioxidant activity (45.49%), antibacterial activity against *S. aureus* and *E. coli*. Biodegradable and eco-friendly	The enhanced tensile strength, reduced solubility, and strong antioxidant and antibacterial activities make this film suitable for solid cosmetics such as bars, sticks, and compact skincare formats. It may function as an internal antioxidant and antimicrobial liner for emulsions or serums, improving stability without continuous water exposure. Its biodegradability aligns well with sustainable packaging initiatives.	[350]
Fish scale gelatin (FSG) and sodium alginate (SA)	Carvacrol-loaded ZIF-8 nanoparticles (CV@ZIF-8)	Solvent casting method	Improved UV-light blocking, elongation at break (+20.86%), reduced water solubility (−1.85×), reduced WVP (−19%), enhanced thermal stability. Exhibited strong antioxidant activity (92.35% DPPH scavenging) and sustained antibacterial effect against *E. coli* and *S. aureus* due to slow release of carvacrol. Extended strawberry shelf life by 8 days.	With improved UV-blocking, reduced solubility, and sustained antimicrobial release, this film is suitable for solid or semi-solid cosmetics prone to oxidation or microbial spoilage. It can be used as a functional insert for natural emulsions requiring slow release of preservative agents. Its partial water sensitivity still limits direct use as a primary packaging layer for liquid formulations.	[351]
Fish gelatin and carrageenan	Turmeric essential oil (TEO) encapsulated in zein nanoparticles (ZNP)	Solvent casting method	Improved physicochemical and mechanical properties; sustained release of TEO; strong antimicrobial activity against *Salmonella enterica*; reduced bacterial load from 6.66 to 2.81 log CFU/g after 14 days; enhanced stability and bioactivity due to ZNP incorporation.	The composite film, featuring sustained release of turmeric essential oil and improved multidimensional stability, is suitable for solid and semi-solid cosmetics needing antimicrobial protection. It may serve as an internal antimicrobial and antioxidant layer for creams and emulsions. Water sensitivity still restricts its use as direct-contact packaging in fully aqueous formulations.	[352]
Fish gelatin and soluble soybean polysaccharide	Tea polyphenols	Solvent casting method	Enhanced antioxidant activity, UV protection, and lipid oxidation inhibition; modified flexibility and surface hydrophilicity; amorphous film structure improving mechanical and optical properties.	This UV-protective, antioxidant film is ideal for packaging light-sensitive solid or semi-solid cosmetics, including balms, makeup pans, and high-oil formulations. Its enhanced bioactivity supports use as an internal protective liner for oxidation-prone emulsions. Nonetheless, its hydrophilic character limits direct exposure to high-moisture cosmetics.	[98]
Fish scale gelatin/dialdehyde alginate	Carbon dots derived from grapefruit peel	Solvent casting method	Enhanced mechanical strength, UV-blocking, water vapor and moisture resistance, fluorescence, and thermal stability; strong antioxidant activity (91.7% DPPH, ~100% ABTS) and antimicrobial activity against bacteria and fungi; extended strawberry shelf life by 7 days.	The film’s enhanced mechanical strength, UV-blocking activity, and moisture resistance make it suitable for solid cosmetics and anhydrous skincare formulations. Its potent antioxidant and antimicrobial properties support use as an intelligent internal liner for natural emulsions vulnerable to microbial and oxidative degradation. However, gelatin-derived sensitivity to water limits primary-contact usage for liquid systems.	[290]
Fish gelatin	Cinnamaldehyde and sulfobutyl ether-β-cyclodextrin inclusion complex	Solvent casting method	Improved elongation at break and light barrier properties; strong antibacterial activity against *Pseudomonas aeruginosa* (98.4% inhibition initially, 82.9% at 72 h); prolonged microbial inhibition and protein preservation during storage; environmentally friendly formulation.	This controlled-release antimicrobial film is appropriate for solid or semi-solid cosmetics requiring prolonged microbial protection, such as balms, sticks, or pressed skincare products. For creams and hydrous emulsions, it may serve as a long-acting antimicrobial insert that maintains formulation integrity. Its moderate water sensitivity limits use as direct-contact packaging material for aqueous products.	[353]
Fish skin gelatin	Mangrove leaf extracts from *Bruguiera gymnorhiza* and *Sonneratia alba*	Solvent casting method	Improved elongation (16.9–19.4%) and water vapor transmission (13.3–13.6 g/m^2^); strong antioxidant activity (12–61%, concentration-dependent); mild antibacterial effect; thickness and tensile strength not significantly affected.	This antioxidant film is suited for oil-based cosmetics, makeup pans, and solid formulations that benefit from protection against oxidative degradation. Although water vapor transmission is moderate, it can act as an internal antioxidant liner for natural emulsions. Direct-contact use with high-moisture products remains limited due to gelatin’s intrinsic hydrophilicity.	[354]
Fish gelatin	Aloe vera gel (AV)	Solvent casting	Addition of AV increased hydrophilicity and solubility; improved thermal and thermo-oxidative stability (higher onset degradation temperature); preserved transparency and homogeneity; showed antioxidant activity (high total phenolic content and radical scavenging activity); exhibited antimicrobial effect against *S. aureus*; FTIR confirmed intermolecular interactions and enhanced Tg due to hydrogen bonding.	Although this composite film offers strong antioxidant and antimicrobial properties suitable for stabilizing natural cosmetic actives, its markedly increased hydrophilicity and solubility restrict direct application to aqueous or gel-based formulations. It is best suited for solid cosmetics or as an internal bioactive liner providing antioxidant protection without prolonged water exposure. Transparency and biocompatibility also make it attractive for eco-friendly solid-product wraps.	[244]
Fish gelatin	Ball-milled tara gum (TG)	Solvent casting method	Film morphology and density influenced by TG particle size and glycerol content; ball-milled TG improved homogeneity, thermal stability, and mechanical strength; optimal plasticizer content (≤20 wt%) enhanced flexibility without compromising performance; SEM, AFM, and FTIR confirmed morphological and structural modifications linked to improved film performance.	This biodegradable film, with improved mechanical behavior and tunable flexibility, is suitable for solid cosmetics, facial mask matrices, or dehydrated skincare products requiring structural stability and controlled hydration. While hydrophilic, its enhanced density and compactness allow short-term contact with mildly hydrated formulations, but it remains unsuitable for direct packaging of liquid cosmetics.	[289]
Kafirin	Citral and quercetin	Solvent casting method	Increased flexibility, reduction in maximum stress and stiffness, increase in strain at break, development of yellow coloration. Antimicrobial activity against viable counts in chicken fillets; antioxidant activity through inhibition of lipid oxidation and reduction of TBARS.	The enhanced antioxidant and antimicrobial performance makes this hydrophobic prolamin-based film ideal for solid and semi-solid cosmetics susceptible to oxidation or microbial growth, such as solid creams, balms, and oil-rich formulations. Its low water sensitivity allows safer interaction with moderately hydrated systems compared to polysaccharide- or gelatin-based films.	[176]
Kafirin	Citral and quercetin	Solvent casting method	Decrease in tensile strength and increase in elongation at break with citral incorporation; reduction in oxygen permeability and water vapor transmission rate; yellow coloration of films. Strong antimicrobial activity against *Campylobacter jejuni*, *Listeria monocytogenes* and *Pseudomonas fluorescens*.	Reduced oxygen and water-vapor permeability, together with potent antimicrobial activity, support applications in semi-solid or oil-based cosmetics that require oxidative and microbial stabilization. Its hydrophobic nature provides better resistance to moisture than chitosan or gelatin films, enabling use as direct-contact packaging for moderately hydrated emulsions.	[110]
Kafirin and polyethylene oxide (PEO) nanocomposite	Red beet extract (natural pH-sensitive pigment)	Electrospinning method	High color stability and reversibility to pH changes (1–10), temperature, and environmental conditions; reversible color transitions; reusable without loss of efficiency; sensitive to aqueous environments due to PEO solubility.	This pH-sensitive, chromatically responsive film is suitable for smart cosmetic packaging aimed at detecting oxidation or pH changes in emulsions, serums, or natural creams. Due to PEO solubility, it should not serve as primary packaging for liquid cosmetics but is effective as an internal diagnostic layer or indicator strip within secondary packaging.	[355]
Kafirin	Quercetin	Solvent casting method (followed by electron beam (EB) irradiation curing)	EB irradiation enhanced mechanical and thermal stability; reduced water vapor permeability, solubility, and transparency; formation of dense crosslinked structure; inhibited microbial growth and oxidative degradation during storage.	Its enhanced mechanical and thermal stability, coupled with reduced solubility and permeability, make this crosslinked film suitable for solid and semi-solid cosmetics, especially those sensitive to oxidation. The hydrophobicity gained through EB irradiation allows limited contact with emulsions, although prolonged exposure to high-water systems should still be avoided.	[356]
Kafirin protein blended with polycaprolactone (PCL)	Carnosic acid (CA)	Electrospinning method	Formation of flexible, hydrophilic fibrous mats with tunable mechanical strength and swelling; hydrogen bonding between kafirin and PCL; controlled release of CA via diffusion; structural stability due to PCL crystalline domains.	This fibrous mat enables controlled antioxidant release and offers moderate water resistance, making it suitable for semi-solid cosmetics requiring slow diffusion of actives (e.g., antioxidant patches, solid balms, or topical delivery matrices). Its stability derives from PCL crystalline domains, supporting safe use near moderately hydrated formulations but not fully aqueous ones.	[357]
Kafirin protein	Glutaraldehyde	Solvent casting method	Microparticle-cast films (<50 μm) exhibited greater water stability than conventional kafirin films. Glutaraldehyde treatment increased tensile strength up to 43%, maintained structural integrity despite plasticizer loss, and enhanced covalent crosslinking between amino and carbonyl groups.	The high water stability achieved through covalent crosslinking renders this film suitable for direct packaging of semi-solid cosmetics or anhydrous pastes that require moisture resistance and mechanical robustness. Its enhanced integrity supports applications where controlled exposure to hydration occurs, though full immersion or high-water emulsions still require caution.	[127]
Kafirin and gelatin	None (focus on structural and barrier design)	Solvent casting method	Distinct multilayer morphology with defined interfaces; intermolecular interactions confirmed by FTIR; enhanced UV protection, mechanical strength, and transparency; asymmetric water vapor barrier—kafirin side more moisture-resistant while gelatin side retains humidity; surface hydrophilicity tunable by layer orientation.	This asymmetric multilayer structure is ideal for cosmetics requiring directional moisture control—such as solid skincare products, pressed powders, or semi-solid formulations that benefit from a hydrophobic external layer and a humidity-retaining internal surface. UV protection and mechanical strength make it useful for formulations containing photosensitive or oxidative-sensitive actives.	[358]
Wheat gluten/zein composite	Ferulic acid and TiO_2_ nanoparticles	Electrospinning method	Enhanced hydrophobicity, mechanical strength, and thermal stability; improved barrier performance; reduced water vapor transmission rate. Antimicrobial effect and ethylene scavenging capacity.	This hydrophobic, thermally stable composite is well suited for semi-solid and oil-based cosmetics that require strong oxidative and UV protection. Its low water vapor transmission rate allows safe contact with moderately hydrated formulations while offering a durable barrier against microbial and oxidative degradation in natural cosmetics.	[359]
Zein	Theaflavins.	Electrospinning method	Improved hydrophobicity and thermo-mechanical stability; alterations in secondary and crystalline structure. Antimicrobial activity against *S. aureus* and *Salmonella paratyphi B*; antioxidant effect with reduced TBARS in meat.	This hydrophobic zein-based film, reinforced with antioxidant theaflavins, is suitable for packaging solid or oil-rich cosmetics requiring oxidative protection and antimicrobial functionality. Its moisture resistance favors applications involving anhydrous balms, pigmented cosmetics, and solid skincare bars. Due to its limited water tolerance, it is better suited for products with low hydration rather than aqueous emulsions.	[360]
Zein	TiO_2_ nanotube arrays (700 nm–5.2 µm)	Solvent casting method	Increased mechanical strength and water resistance; intermolecular hydrogen bonding with zein matrix. Antimicrobial activity against *E. coli* under UV irradiation.	The increased mechanical strength and water resistance make this composite useful for semi-solid cosmetics that benefit from enhanced stability and UV protection. Its hydrophobic profile supports direct contact with moderately hydrated formulations, while the photoactive antimicrobial response offers added value for packaging of natural creams or gel-like products stored under ambient light.	[361]
Zein	*Foeniculum vulgare* and *Carum carvi* essential oils.	Electrospinning method	Mechanical strength in the range of 6–9 MPa; low water vapor permeability; molecular interactions confirmed by FTIR. Antimicrobial activity against *S. aureus*, *L. monocytogenes*, and *Y. enterocolitica*.	Low water-vapor permeability and strong antimicrobial activity make this system appropriate for solid and semi-solid cosmetics with microbial sensitivity, such as natural deodorants, balms, and essential oil–based formulations. Its hydrophobic matrix prevents excessive moisture absorption, supporting improved stability of moderately hydrated products but not fully aqueous systems.	[362]
Zein	Peppermint oil with methyltriethoxysilane coating	Electrospinning method	High hydrophobicity (water contact angle 138°); controlled release of peppermint oil; stable structural properties. Antimicrobial activity against *S. aureus*; antioxidant activity; extension of pork shelf-life by 100%.	This highly hydrophobic and structurally stable film is suitable for solid cosmetics requiring fragrance retention or controlled volatile release, such as solid perfumes or aromatherapeutic balms. Although it offers antimicrobial activity, its water-repellent nature limits use with aqueous emulsions, making it ideal primarily for anhydrous or low-moisture products.	[363]
Zein, polyvinyl alcohol and chitosan	Anthocyanin extract	Solvent casting method	Enhanced interfacial compatibility and flexibility; improved mechanical strength (elongation at break 68.7%, elastic modulus 187.2 MPa); excellent UV protection (200–350 nm) and reduced water vapor permeability (6.60 × 10^−11^ g·m·m^−2^·s^−1^·Pa^−1^); antioxidant activity (45.8%) and colorimetric response (ΔE = 20.2) to volatile amines.	This intelligent, UV-protective, antioxidant composite is well-suited for semi-solid or oil-containing cosmetic formulations that benefit from oxidation monitoring and barrier reinforcement. While the presence of PVA and chitosan introduces some hydrophilicity, the reduced WVP supports short-term contact with mildly hydrated products, though it remains unsuitable for high-water emulsions as primary packaging.	[240]
Zein and poly(lactic acid) (PLA) composite fibrous films	Carvacrol	Electrospinning method	Successful encapsulation of carvacrol; smooth bead-free fibers; enhanced antioxidant activity (62–75% for zein fibers, 53–65% for PLA fibers); sustained diffusion-controlled release; antimicrobial inhibition of mold and yeast (99.6% for zein, 91.3% for PLA); improved thermal stability in PLA fibers.	These fibrous mats are ideal for solid and semi-solid cosmetics requiring antimicrobial and antioxidant protection, such as masks, balms, or natural creams. The hydrophobicity of zein and PLA supports controlled release while maintaining structural integrity in moderately hydrated formulations. However, they are not recommended for direct packaging of high-moisture products.	[364]
Cassava starch and chitosan	Lemongrass essential oil (LEO)	Solution casting method	Optimal physical and mechanical properties at 20% glycerol; antimicrobial activity of LEO against *E. coli*, *S. typhimurium*, *S. aureus*, *B. cereus*, *C. albicans*, and *A. niger*; reduced water vapor permeability (WVP) and solubility; effective in reducing microbial growth and extending chili shelf life to 16 days.	Antimicrobial and antioxidant film suitable for solid or semi-solid cosmetic products (bars, balms, oil-based creams); may serve as an inner antimicrobial sachet within containers for natural emulsions, but not intended for direct long-term contact with high-moisture formulations.	[365]
Maize starch	Nisin and Natamycin	Solution casting method	Exhibited strong antimicrobial activity against *Bacillus cereus* and *Aspergillus niger*; synergistic effect with modified atmosphere packaging (MAP: N_2_/CO_2_ 70:30) extended the shelf life of dairy-based confection from 21 to 42 days; maintained physicochemical, microbiological, and sensory quality.	Active antimicrobial coating or insert for protecting preservative-free cosmetic creams and emulsions from fungal or bacterial contamination; recommended as a secondary or internal element rather than a primary packaging layer in contact with liquid formulations due to the hydrophilic nature of starch.	[366]

Abbreviations: DPPH: 2,2-diphenyl-1-picrylhydrazyl; ABTS: 2,2′-azino-bis(3-ethylbenzothiazoline-6-sulfonic acid); FTIR: Fourier Transform Infrared Spectroscopy; SEM: Scanning Electron Microscopy; XRD: X-ray Diffraction; WVTR: Water Vapor Transmission Rate; OP: Oxygen Permeability; UV–VIS: Ultraviolet–Visible spectroscopy; CS: Chitosan; CFS: Cell-Free Supernatant; WRE: Watermelon Rind Extract; SFE: Scallion Flower Extract; ZnO@gal: Zinc Oxide Nanoparticles loaded with Gallic Acid; PCL: Poly(ε-caprolactone); UGNP: Urchin-like Gold Nanoparticles; ɛ-PL: ɛ-Polylysine; EuNE: Eugenol Nanoemulsion; AVG: Aloe vera Gel; ZnONP: Zinc Oxide Nanoparticles; HASP: Hydroxyapatite Nanoparticles; HANP: Hydroxyapatite Nanoparticles; Tg: Glass Transition Temperature; XPS: X-ray Photoelectron Spectroscopy; AFM: Atomic Force Microscopy; TS: Tensile Strength; WVP: Water Vapor Permeability; OP: Oxygen Permeability; TBARS: Thiobarbituric Acid Reactive Substances; ZIF-8: Zeolitic Imidazolate Framework-8; CV@ZIF-8: Carvacrol-loaded ZIF-8 Nanoparticles; ZNP: Zein Nanoparticles; CFU: Colony Forming Units; AV: Aloe vera; TG: Tara Gum; EB: Electron Beam; CA: Carnosic Acid; PEO: Polyethylene Oxide; PLA: Poly(lactic acid); LEO: Lemongrass Essential Oil; MAP: Modified Atmosphere Packaging; SA: Sodium Alginate; FSG: Fish Scale Gelatin; FLE: *Ficus carica* Leaf Extract; TE: Turmeric Essential Oil; TEO: Thyme Essential Oil; CA: Carnosic Acid; N_2_/CO_2_: Nitrogen/Carbon Dioxide mixture.

## Data Availability

No new data were created in this study and therefore, data sharing is not applicable to this article.
